# Publisher Correction: GWAS of bone size yields twelve loci that also affect height, BMD, osteoarthritis or fractures

**DOI:** 10.1038/s41467-019-10425-4

**Published:** 2019-05-24

**Authors:** Unnur Styrkarsdottir, Olafur A. Stefansson, Kristbjorg Gunnarsdottir, Gudmar Thorleifsson, Sigrun H. Lund, Lilja Stefansdottir, Kristinn Juliusson, Arna B. Agustsdottir, Florian Zink, Gisli H. Halldorsson, Erna V. Ivarsdottir, Stefania Benonisdottir, Hakon Jonsson, Arnaldur Gylfason, Kristjan Norland, Katerina Trajanoska, Cindy G. Boer, Lorraine Southam, Jason C. S. Leung, Nelson L. S. Tang, Timothy C. Y. Kwok, Jenny S. W. Lee, Suzanne C. Ho, Inger Byrjalsen, Jacqueline R. Center, Seung Hun Lee, Jung-Min Koh, L. Stefan Lohmander, Lan T. Ho-Pham, Tuan V. Nguyen, John A. Eisman, Jean Woo, Ping-C. Leung, John Loughlin, Eleftheria Zeggini, Claus Christiansen, Fernando Rivadeneira, Joyce van Meurs, Andre G. Uitterlinden, Brynjolfur Mogensen, Helgi Jonsson, Thorvaldur Ingvarsson, Gunnar Sigurdsson, Rafn Benediktsson, Patrick Sulem, Ingileif Jonsdottir, Gisli Masson, Hilma Holm, Gudmundur L. Norddahl, Unnur Thorsteinsdottir, Daniel F. Gudbjartsson, Kari Stefansson

**Affiliations:** 1 deCODE genetics/Amgen Inc., Reykjavik, 101 Iceland; 2 Faculty of Medicine, University of Iceland, Reykjavik, 101 Iceland; 3 Department of Epidemiology, ErasmusMC, 3015 GD Rotterdam, The Netherlands; 4 Department of Internal Medicine, ErasmusMC, 3015 GD Rotterdam, The Netherlands; 5 Wellcome Trust Sanger Institute, Hinxton, CB10 1SA UK; 6 Wellcome Trust Centre for Human Genetics, University of Oxford, Oxford, OX3 7BN UK; 7 Jockey Club Centre for Osteoporosis Care and Control, Faculty of Medicine, The Chinese University of Hong Kong, Hong Kong, China; 8 Faculty of Medicine, Department of Chemical Pathology and Laboratory for Genetics of Disease Susceptibility, Li Ka Shing Institute of Health Sciences, The Chinese University of Hong Kong, Hong Kong, China; 9 CUHK Shenzhen Research Institute, Shenzhen, 518000 China; 10 Department of Medicine and Therapeutics, Prince of Wales Hospital, Hong Kong, China; 11 Faculty of Medicine, Department of Medicine and Therapeutics, The Chinese University of Hong Kong, Hong Kong, China; 12 Department of Medicine, Alice Ho Miu Ling Nethersole Hospital and Tai Po Hospital, Hong Kong, China; 13 JC School of Public Health and Primary Care, Faculty of Medicine, The Chinese University of Hong Kong, Hong Kong, China; 14 Nordic Bioscience A/S, 2730 Herlev, Denmark; 15 Bone Biology Division, Garvan Institute of Medical Research, Sydney, NSW 2010 Australia; 16 School of Medicine Sydney, University of Notre Dame Australia, Sydney, NSW 2010 Australia; 17 St Vincent’s Clinical School, University of New South Wales, Sydney, NSW 2010 Australia; 18 Division of Endocrinology and Metabolism, Asan Medical Center, University of Ulsan College of Medicine, Seoul, 05505 Korea; 19 Orthopaedics, Department of Clinical Sciences Lund, Lund University, SE-22 100 Lund, Sweden; 20 Bone and Muscle Research Lab, Ton Duc Thang University, Ho Chi Minh City, 700000 Vietnam; 21 School of Biomedical Engineering, University of Technology Sydney, Sydney, NSW 2007 Australia; 22 Clinical Translation and Advanced Education, Garvan Institute of Medical Research, Sydney, NSW 2010 Australia; 23 Institute of Chinese Medicine, The Chinese University of Hong Kong, Hong Kong, China; 24 Institute of Genetic Medicine, Newcastle University, Newcastle-upon-Tyne, NE1 7RU UK; 25 Institute of Translational Genomics, Helmholtz Zentrum München, 85764 München, Germany; 26 Department of Emergengy Medicine, Landspitali, The National University Hospital of Iceland, 101 Reykjavik, Iceland; 27 Research Institute in Emergency Medicine, Landspitali, The National University Hospital of Iceland, and University of Iceland, 101 Reykjavik, Iceland; 28 Department of Medicine, Landspitali–The National University Hospital of Iceland, 101 Reykjavik, Iceland; 29 Department of Orthopedic Surgery, Akureyri Hospital, 600 Akureyri, Iceland; 30 Institution of Health Science, University of Akureyri, 600 Akureyri, Iceland; 31 Research Service Center, Reykjavik, 201 Iceland; 32 Department of Endocrinology and Metabolism, Landspitali, The National University Hospital of Iceland, 101 Reykjavik, Iceland; 33 Department of Immunology, Landspitali–The National University Hospital of Iceland, 101 Reykjavik, Iceland; 34 School of Engineering and Natural Sciences, University of Iceland, Reykjavik, 107 Iceland

**Keywords:** Gene expression analysis, Genome-wide association studies, Osteoarthritis, Bone

Correction to: *Nature Communications* 10.1038/s41467-019-09860-0, published online 3 May 2019.

The original HTML version of this Article was updated shortly after publication to add links to the Peer Review file.

In addition, affiliations 16 and 17 incorrectly read ‘School of Medicine Sydney, University of Notre Dame Australia, Sydney, WA, 6160, Australia’ and ‘St Vincent’s Clinical School, University of New South Wales Medicine, University of New South Wales, Sydney, NSW, 2052, Australia.’ This has now been corrected in both the PDF and HTML versions of the Article.

