# Supplementary material for: GWAS of bone size yields twelve loci that also affect height, BMD, osteoarthritis or fractures
Source: Nat Commun. 2019 May 3;10:2054. doi: 10.1038/s41467-019-09860-0 (PMC6499783; doi:10.1038/s41467-019-09860-0)
Supplement: Supplementary file 1 — Supplementary Information [file 41467_2019_9860_MOESM1_ESM.pdf]

Styrkarsdottir, *et.al*. Supplementary Information: GWAS of bone size finds twelve loci that also affect height, BMD, osteoarthritis or fractures

Table of Contents

SUPPLEMENTARY NOTES 4

Supplementary Note 1. MIR196A2 4

Supplementary Note 2. Hip fractures and DXA area, BMD, and BMC 7

Supplementary Note 3. arcOGEN consortium investigators 8

Supplementary Note 4. Acknowledgement 8

SUPPLEMENTARY FIGURES. 10

Supplementary Figure 1. Images of bone areas from DXA scans 10

Supplementary Figure 2. Locus plots of genome wide significant associations 11

Supplementary Figure 3. Effect of rs11614913 on mir-196-a2 15

Supplementary Figure 4. Fraction of genes with predicted miR-196a-5p 3´UTR recognition sites that have experimental support 16

Supplementary Figure 5. The effect of the rs11614913 variant in MIR196A2 on target gene expression in human HEK293 cell line 17

Supplementary Figure 6. Downregulation in MIR196A2 insert cells for genes with predicted 3'UTR recognition sites. 18

Supplementary Figure 7. Gene set enrichment for the entire set of genes suppressed by MIR196A insert. 19

Supplementary Figure 8. Correlation between rs11614913 and rs371683123 at 12q13.13 and expression of the *HOXC8* gene in adipose tissue. 20

Supplementary Figure 9. Correlation between effects of GIANT height variants on adult height and effects on DXA areas 21

Supplementary Figure 10. Correlation between effects of GEFOS BMD variants on BMD and effects on DXA areas 22

Supplementary Figure 11. Gating strategy to sort GFP positive HEK293T cells 23

Supplementary Tables 24

Supplementary Table 1. Conditional analysis between variants at three loci in the Icelandic discovery dataset: a) between two variants at 15q25.2 at the *ADAMTSL3* and *SH3GL3* genes, b) between the two variants at the *SOX9* locus that associate with intertrochanteric area in the Icelandic discovery dataset and two recently published variants that associate with hip shape measures, and c) between the 4q31.21 Icelandic variant and a recently published variant associated with hip shape measure 24

Supplementary Table 2. The number of individuals in all participating studies split by phenotype26

Supplementary Table 3. Gene function analysis. A) Analysis of gene ontology overrepresentation for the high-confidence miR-196a-5p target genes as carried out in Panther (pantherdb.org); showing results at FDR < 5% ordered according to decreasinging level of overrepresentation ratios. B) The seventeen miR-196a-5p target genes listed out with mean expression in HEK293T (inhouse data) and osteoblasts (Fantom5 data). 27

Supplementary Table 4. Differential expression of miR-196a-5p target genes in pairwise comparisons for gene expression experiments carried out in cells transfected with MIR196A2 C-allele insert, T-allele insert and empty vector control 29

Supplementary Table 5. Expression correlation between HOXC8 and rs11614913 and rs371683123 before and after conditioning on the other variant 32

Supplementary Table 6. Association of DXA area markers with vertebral fractures in all sample-sets combined 33

Supplementary Table 7. Association of height markers and BMD markers with DXA area measures in Iceland 34

Supplementary Table 8. Mendelian Randomization 38

Supplementary Table 9. Genetic correlations between DXA area, DXA BMD, hip or knee osteoarthritis and hip fractures 40

Supplementary Table 10. Contribution of DXA area, BMC and BMD to the risk of hip fractures, full model 43

Supplementary References: 44

SUPPLEMENTARY NOTES

Supplementary Note 1. MIR196A2

The mature miRNA (miR) sequence derived from MIR196A2 is excised from the 5´ arm of the pre-miR hairpin, then referred to as miR-196a-5p (mirbase.org). This microRNA is tissue-specific and is enriched in chondrocytes and in glycosaminoglycan secreting cells (chondrocytes, tendon cells, fibroblasts) (http://fantom.gsc.riken.jp). The rs11614913 variant resides on the 3´ arm (Supplementary Fig. 3) and for this reason does not alter the seed sequence. Therefore, the variant is not expected to affect recognition of it´s target gene mRNAs. However, the T-allele in the mature miR duplex introduces a wobble bond, i.e. pairing between uracil and guanine in RNA, at the 5´end of the 5p-arm (Supplementary Fig. 3). On the basis of this observation, we hypothesized that the effect of the variant might lie in efficiency, rather than specificity, of target gene repression due to either 1) differences in thermodynamic stability of the mature miR duplex by genotype thereby leading to effects on incorporation of the mature 5p strand into Ago complex [1] or 2) effects of the genotype on miR processing to the mature miR duplex form [2,3], as previously proposed [4], which could affect the abundance of the mature miR duplex. In both cases, we expect the variant to have a general effect on transcription levels accross genes targeted for repression by miR-196a-5p.

In order to assess the impact of the rs11614913 variant in MIR196A2 on target gene expression, we carried out an experiment in which we transfected HEK293T cells with over-expression plasmids containing 1) *MIR196A2* with the C-allele, 2) *MIR196A2* with the T-allele and 3) no insert (empty plasmid). In order to avoid biases introduced by variation in transfection efficiency between cell cultures, we carried out cell sorting by flow cytometry for cells marked by GFP protein expression from plasmid DNA. After cell sorting we isolated RNA and carried out RNA-seq (polyA+) to assess genome-wide transcript expression levels. The experiments were independently done in six replicates for each condition (n=18).

The RNA-seq transcript expression was quantified with Kallisto [5] making use of Ensembl transcript sequences (version 87) as a reference transcriptome. Gene expression was computed by aggregating expression across transcripts. We then used DESeq2 for Bioconductor in R to analyse differential expression [6]. In principle, the biological interpretation of differentially expressed genes in our set up can be seen as 1) primary effects and 2) secondary effects. Here, the primary effects are the direct target genes of mir-196a2 whereas the secondary effects include downstream effects caused by downregulation of the target genes, e.g. downregulation of transcription factors (TFs) by miR-196a2 can lead to downstream changes in gene expression. In order to study the genotype effects on the primary target genes, we sought to define a group of high-confidence miR-196a-5p target genes. We

downloaded a list of 302 predicted target genes (TargetScan 7.2) and we find that target genes with high conservation scores (P_{CT}; Probability of conserved targeting), defined in TargetScan 7.2, are more likely to have experimental support (genes identified as plausible direct targets of miR-196a-5p; according to Tarbase V8[7]) as compared with those with low conservation score (Supplementary Fig. 4). In particular, the fraction of genes containing a 3´UTR recognition site, predicted by TargetScan v7.2, with experimental support in Tarbase starts to become particularily high at $P_{CT} > 0.86$ (Supplementary Fig. 4). On the basis of this observation, we defined our high confidence miR-196a-5p target genes as genes with *in silico* predicted 3´UTR recognition site for miR-196a-5p where the P_{CT} score is > 0.86; resulting in a list of seventeen genes. This P_{CT} score translates into less than 14% probability for the miR-196a-5p binding sites in the target genes being conserved by chance. The biological relevance of these genes to human variation in bone morphology is seen in that the seventeen target genes are significantly enriched for functional roles involved in skeletal system development (13.5 fold overrepresentation; *P*-value=2.36e-5), see Supplementary Table 11. Fourteen of the high-confidence miR-196a-5p target genes are expressed in the MIR196A2 transfected HEK293T cells.

To further assess the validity of the defined list of miR-196a-5p target genes, we find that these genes are highly enriched among the set of genes found significantly downregulated in cells with MIR196A2 insert (C- or T-alleles) *versus* empty controls (Supplementary Fig. 5A and B). Specifically, 13 (92.9%) and 12 (85.7%) out of the fourteen expressed target genes are in the group of significantly downregulated genes ($P_{adj} < 0.05$) in C- and T-allele transfected cells, respectively. In contrast, without thresholding the P_{CT} conservation score, the intersection with downregulated genes is considerably lower, i.e. 50% and 40.4% in C- and T-allele transfected cells, respectively. Supplementary Fig. 6 shows the mean and standard deviation in log2 fold-change values (derived from the MIR195A2 insert versus control comparison) accross genes containing *in silico* predicted 3´UTR recognition sites for miR-196a-5p (TargetScan v7.2) at varying P_{CT} score thresholding level. None of the target genes were significantly upregulated.

When comparing MIR196A2 C-allele versus T-allele transfected cells, we observe that all of the fourteen target genes are more down-regulated in C-allele transfected cells (Students‘s t-test P = 0.00016) (Supplementary Fig. 5C); although this effect is clearly subtle as reflected in 1) low effect size log2 fold values and 2) that none of the genes tested individually remain statistically significant after adjusting for multiple testing. Log2 fold change values calculated using the method implemented in DESeq2 v1.16.1 (Bioconductor for R). We also carried out a permutation based test to determine significance; we counted how often the observed difference was lower than the differences derived from permutations of samples agnostic to the allele annotation; $\binom{12}{6}$ =924 possible unique permutations. This analysis demonstrates a significant difference in downregulation of the target genes between the two

transfected cells types (*P* = 0.042) showing that the rs11614913[T] allele is less effective, when compared with the C-allele, in repressing expression of miR-196a-5p target genes.

Finally, in order to gain insight into the biological effects of miR-196a2, we carried out a gene set enrichment analysis for functional categories in the comparison between MIR196A2 insert (C- or T-allele) and empty vector controls. This analysis was carried out using fgsea in Bioconductor for R using gene ontology (GO) terms derived from MSigDB v6.2[ref8] using log2 fold-change values as the gene ranking parameter. The top significant functional category (P-adjusted < 0.05) involves downregulation of genes involved in energy metabolism (e.g. GO terms oxidative phosphorylation and electron transport chain). Also, a functional category relevant to biological processes in bone morphology was found to be enriched (GO term embyonic skeletal system morphogenesis). By looking at the leading edge genes for the embryonic skeletal system morphogenesis category in this analysis, i.e. genes in this category with large effect sizes for downregulation, we note that 7 out of these 21 genes have experimental support as direct target genes of miR-196a-5p. Thus, many of the genes driving the enrichment for this functional category are likely primary targets of miR-196a-5p, but others are not; wheather these are downstream effects caused by miR-196a-5p remains to be fully established.

Supplementary Note 2. Hip fractures and DXA area, BMD, and BMC

We performed a multivariable logistic regression for hip fractures, using age, height and sex adjusted measurements of DXA area, BMD and BMC of lumbar spine, as well as area and BMD of femoral neck, intertrochanteric and trochanteric and BMC of the total hip as explanatory variables. Each of these 10 phenotypes showed independent association with hip fractures, adjusting for the other 9 phenotypes, according to likelihood ratio test (Supplementary Table 17).

Furthermore, five of the loci associated with bone area were also nominally associated with hip fractures ($p<0.05$). The figure below shows the estimated effect sizes, with 95% confidence intervals, of each of these five variants for the phenotypes listed above. There it can be seen that all of these variants also show significant association with several BMD or BMC measurements so we are not able to see whether the increased risk of fractures is due to bone area rather than BMD or BMC.

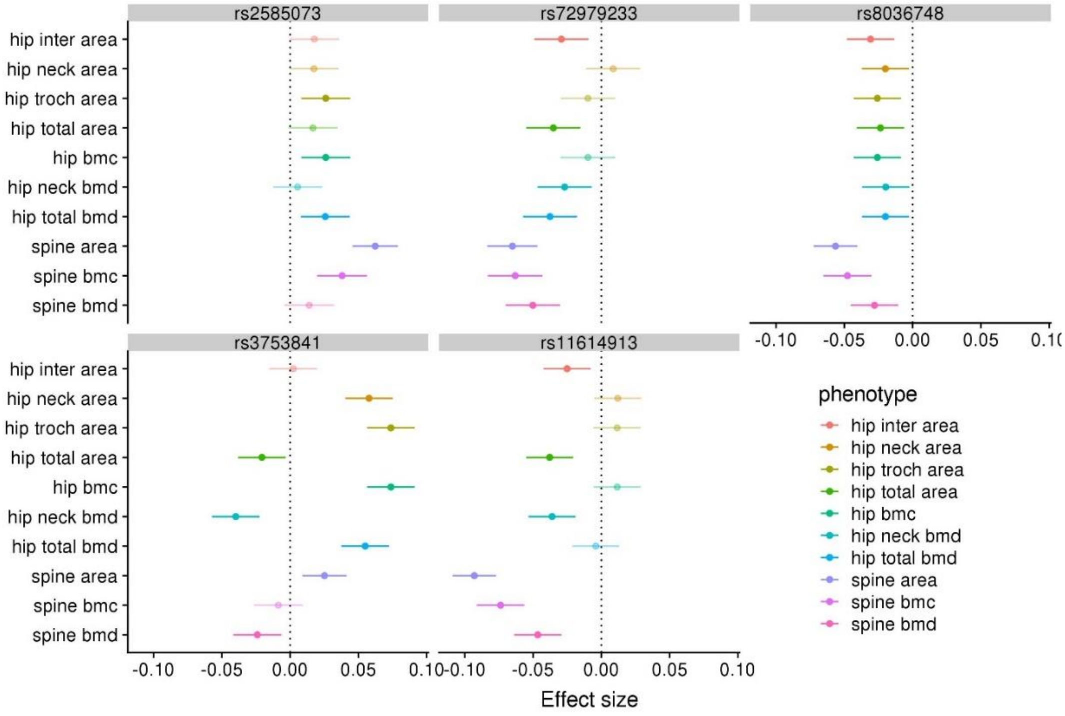

Supplementary Note 3. arcOGEN consortium investigators

John Loughlin (Newcastle University), Nigel Arden (University of Oxford and University of Southampton), Fraser Birrell (Newcastle University), Andrew Carr (University of Oxford), Kay Chapman (University of Oxford), Panos Deloukas (William Harvey Research Institute), Michael Doherty (University of Nottingham), Andrew McCaskie (University of Cambridge), William E. Ollier (University of Manchester), Ashok Rai (University of Worcester), Stuart H. Ralston (University of Edinburgh), Tim D. Spector (King's College London), Ana M. Valdes (University of Nottingham), Gillian A. Wallis (University of Manchester), J. Mark Wilkinson (University of Sheffield), Eleftheria Zeggini (Wellcome Trust Sanger Institute)

Supplementary Note 4. Acknowledgement

We thank the subjects of the Icelandic deCODE study, the Danish PERF study, the Swedish MDC study, the Dutch Rotterdam study, the UK arcOGEN and UK Biobank studies, the Australian Dubbo study, the Chinese Hong Kong Mr OS and Ms OS studies, the Korean AMC study and the Vietnamese Osteoporosis Study.

We thank the staff at deCODE genetics core facilities and the staff at the Research Service Center for their important contribution to this work, and Hjalti Sigurdarson for the artwork.

A part of this research has been conducted using the UK Biobank Resource under Application Number 23359.

The Rotterdam Study is funded by Erasmus Medical Center and Erasmus University, Rotterdam, Netherlands Organization for the Health Research and Development (ZonMw), the Research Institute for Diseases in the Elderly (RIDE), the Ministry of Education, Culture and Science, the Ministry for Health, Welfare and Sports, the European Commission (DG XII), and the Municipality of Rotterdam. The authors are grateful to the study participants, the staff from the Rotterdam Study and the participating general practitioners and pharmacists. The generation and management of GWAS genotype data for the Rotterdam Study (RS I, RS II, RS III) was executed by the Human Genotyping Facility of the Genetic Laboratory of the Department of Internal Medicine, Erasmus MC, Rotterdam, The Netherlands. The GWAS datasets are supported by the Netherlands Organisation of Scientific Research NWO Investments (nr. 175.010.2005.011, 911-03-012), the Genetic Laboratory of the Department of Internal Medicine, Erasmus MC, the Research Institute for Diseases in the Elderly (014-93-015; RIDE2), the Netherlands Genomics Initiative (NGI)/Netherlands Organisation for Scientific Research (NWO) Netherlands Consortium for Healthy Aging (NCHA), project nr. 050-060-810. F.R and K.T are supported by the Netherlands Scientific Organization (NWO) and ZonMW Project number: NW O/ZONMW-VIDI-0 16-136-367. We thank Pascal Arp, Mila Jhamai, Marijn Verkerk, Lizbeth Herrera and Marjolein Peters, MSc, and

Carolina Medina-Gomez, MSc, for their help in creating the GWAS database, and Karol Estrada, PhD, Yurii Aulchenko, PhD, and Carolina Medina-Gomez, MSc, for the creation and analysis of imputed data

arcOGEN: arcOGEN (http://www.arcogen.org.uk/) was funded by a special purpose grant from Arthritis Research UK (grant 18030).

UKHLS: These data are from Understanding Society: The UK Household Longitudinal Study, which is led by the Institute for Social and Economic Research at the University of Essex and funded by the Economic and Social Research Council. The data were collected by NatCen and the genome wide scan data were analysed by the Wellcome Trust Sanger Institute. The Understanding Society DAC have an application system for genetics data and all use of the data should be approved by them. The application form is at: https://www.understandingsociety.ac.uk/about/health/data.

LSL received support from the Swedish Research Council and the Medical faculty of Lund University.

JL received support from Arthritis Research UK (grant 20771), and from The Medical Research Council and Arthritis Research UK as part of the MRC-Arthritis Research UK Centre for Integrated Research into Musculoskeletal Ageing (CIMA, grants JXR 10641 and MR/P020941/1).

For the Dubbo study: The Dubbo Osteoporosis Epidemiology Study was supported by the Australian National Health and Medical Research Council, Grant Number APP1031494 (CIA T. Nguyen). We gratefully acknowledge the assistance of Sr Janet Watters, Donna Reeves, Shaye Field and Jodie Martin for the interview, data collection and measurement bone mineral density. We also appreciate the invaluable help of the staff of Dubbo Base Hospital.

For VOS: The Vietnam Osteoporosis Study was funded by the Foundation for Science and Technology Development of Ton Duc Thang University (FOSTECT, http://fostect.tdt.edu.vn), Grant number FOSTECT.2014.BR.09, and a grant from the Department of Science and Technology of Ho Chi Minh City. We sincerely thank Mrs Tran Thi Ngoc Trang and Fr Pham Ba Lam for coordinating the recruitment of participants. We also thank doctors and medical students of the Pham Ngoc Thach University of Medicine for the data collection and clinical measurements.

J.W. and N.T. have been supported by CUHK VC discretionary fund and The Hong Kong Jockey Club Charities Trust. JSWL received support from Health and Medical Research Fund #12133811.

J-M. K. was supported by grant from the Korea Health Technology R&D Project, Ministry of Health and Welfare, Republic of Korea (Projects HI15C2792).

SUPPLEMENTARY FIGURES.

Supplementary Figure 1. Images of bone areas from DXA scans

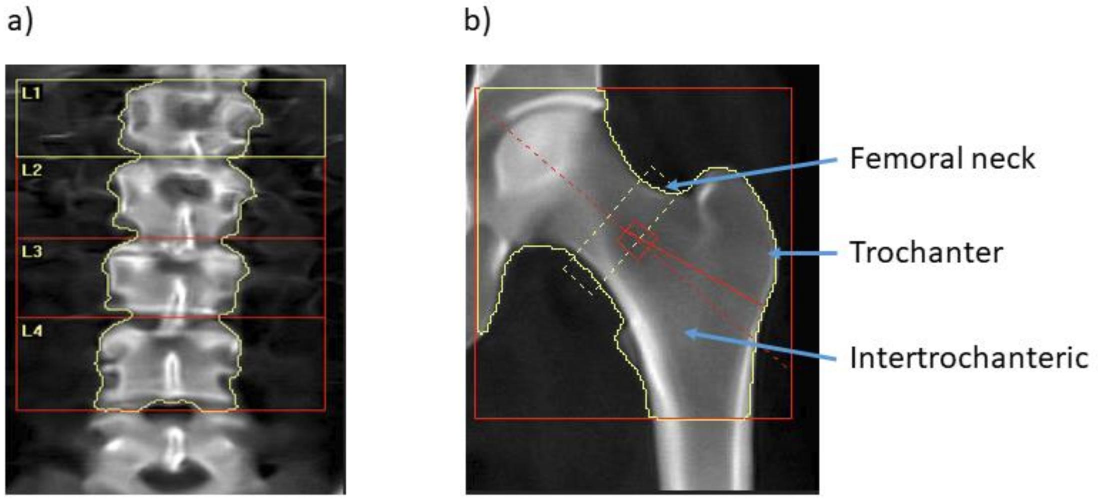

a) Spine vertebrae L1 – L4. **b**) Hip area, showing the femoral neck (within the yellow box), trochanter (defined by the yellow outline, the red solid line and femoral neck line) and intertrocanteric (defined by the red solid line, the femoral neck line and the yellow outline) regions. Total hip area is the sum of femoral neck, trochanter and intertrochanteric areas and does not include the femoral head. The red broken line represent the symmetry axis.

The images are from http://www.hologic.com/horizon-dxa-system-image-gallery

Supplementary Figure 2. Locus plots of genome wide significant associations

P values (-log10) of SNP associations with the respecitve phenotypes in the Icelandic discovery samples are plotted against their chromosomal positions (NCBI Build 38 coordinates). SNPs are colored to reflect their linkage disequilibrium (LD) with the marker that was genotyped in the replication samples, in most cases this is the top marker, however, at some loci a functional single-variant genotyping assay could not be made for the top marker and, therefore, a proxy SNP was used. The blue line indicates recombination rates, based on the Icelandic recombination map for males and females combined, with the peaks indicating recombination hotspots. Known genes in the region are shown underneath the plot. The plots were created using a stand-alone version of LocusZoom software [9]. Source data are provided as a Source Data file.

a) Spine Area (L1-L4) 12q13.13

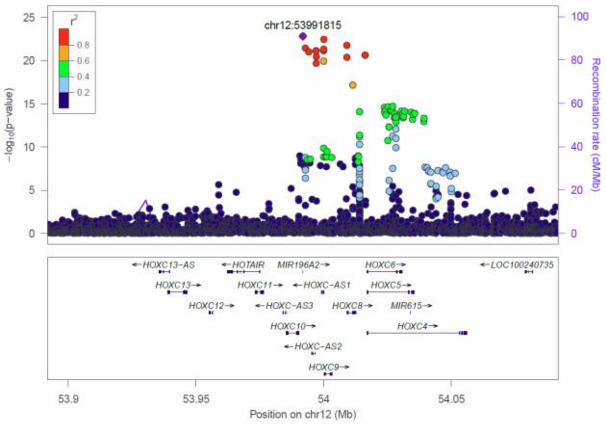

c) Spine area (L1-L4) 1p36.12

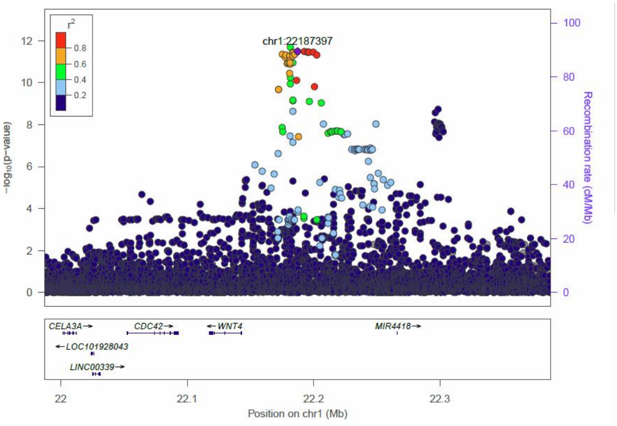

b) Spine area (L1-L4) 20q11.22

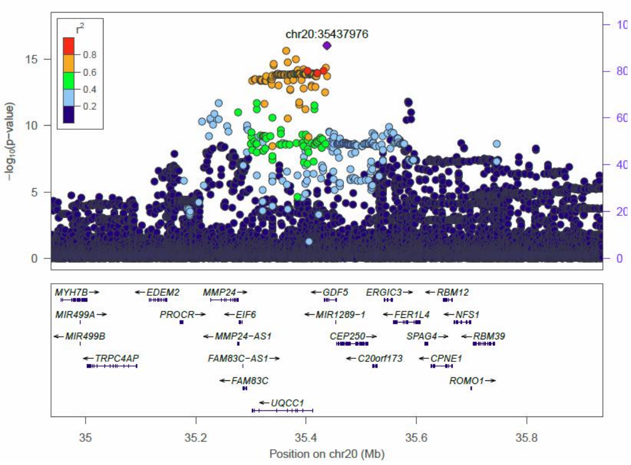

d) Spine area (L1-L4) 18q21.1

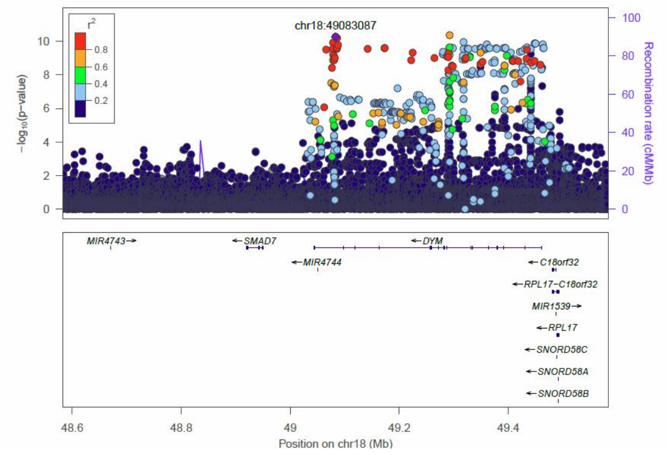

e) Spine area (L1-L4) 15q25.2

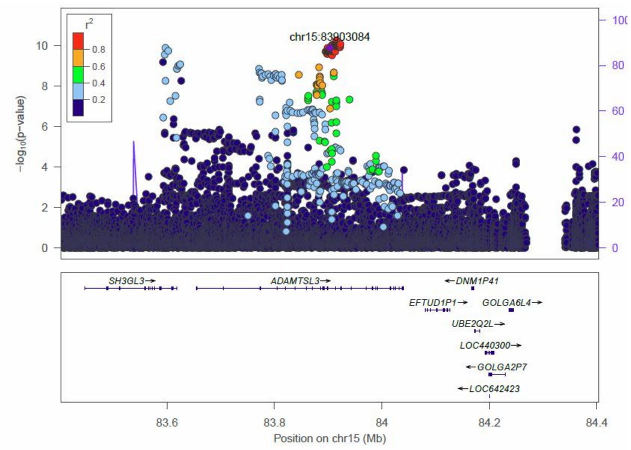

h) Spine area (L1-L4) 11q13.4

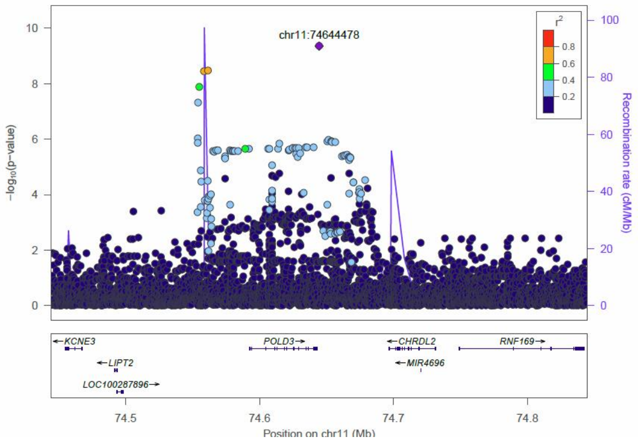

x) Hip total 20q11.22

f) Spine area (L1-L4) 15q25.2_2

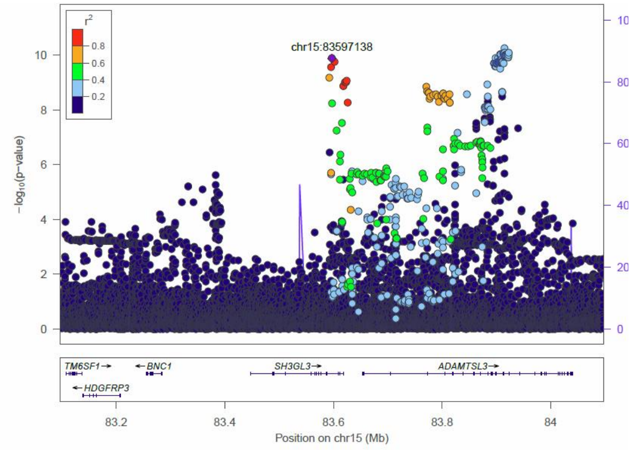

i) Hip total 20q11.22

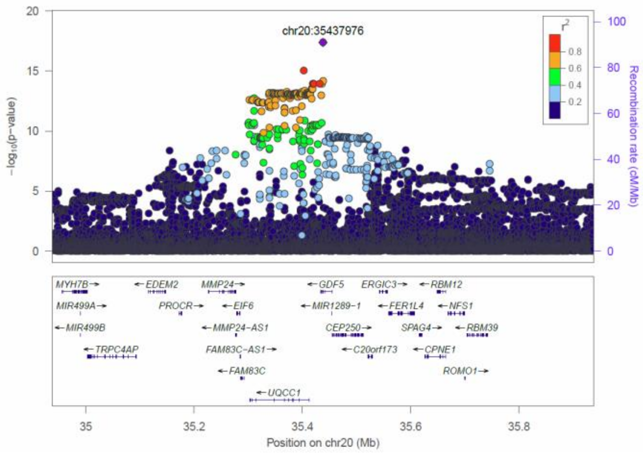

g) Spine area (L1-L4) 6q14.1

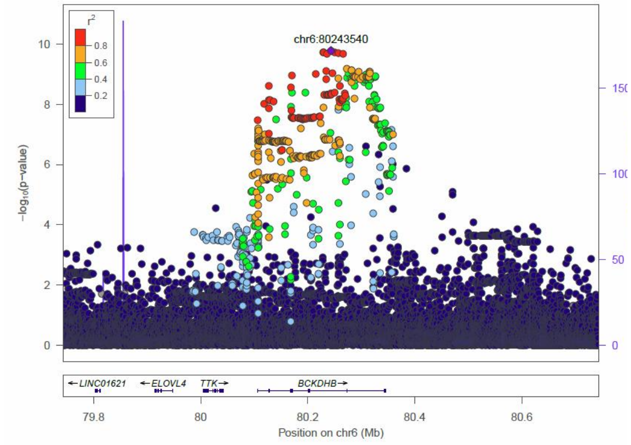

j) Hip total 1p21.1

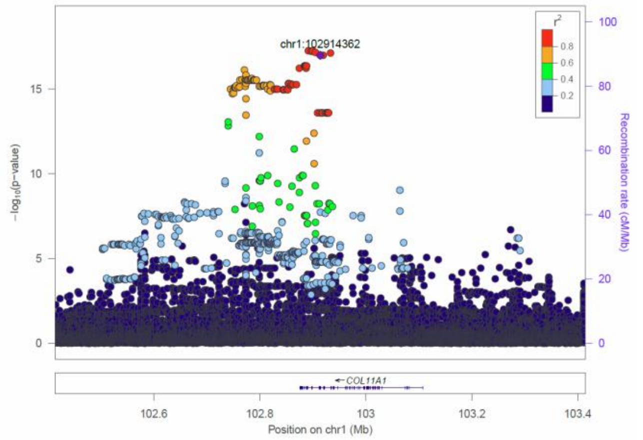

k) Hip total 3p14.3

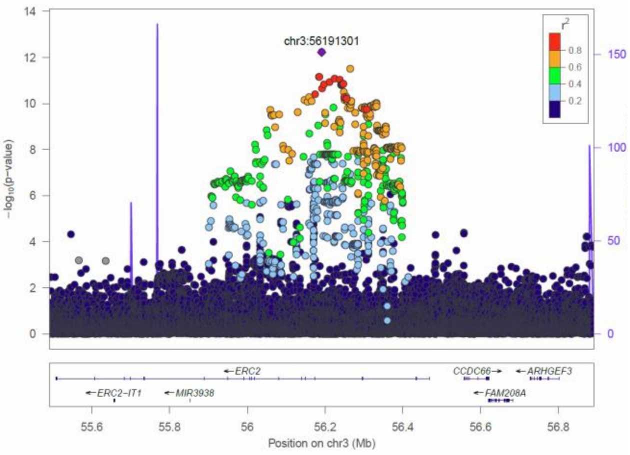

n) Hip femoral neck 4q31.21

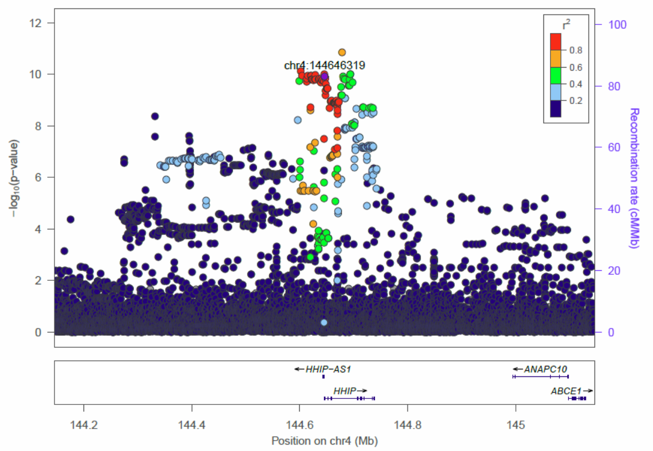

l) Hip total 18q23

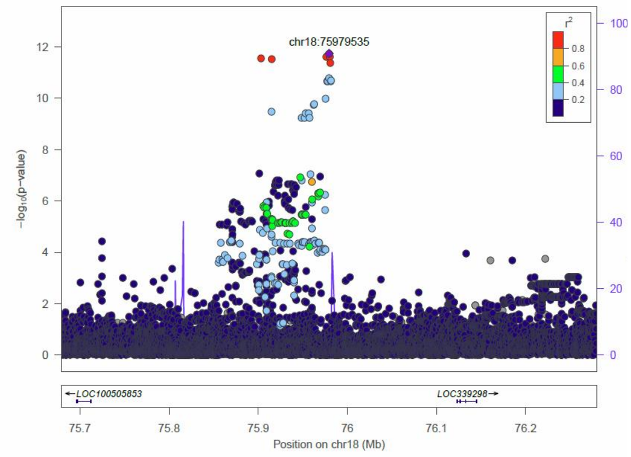

o) Hip intertrochanter area 17q24.3

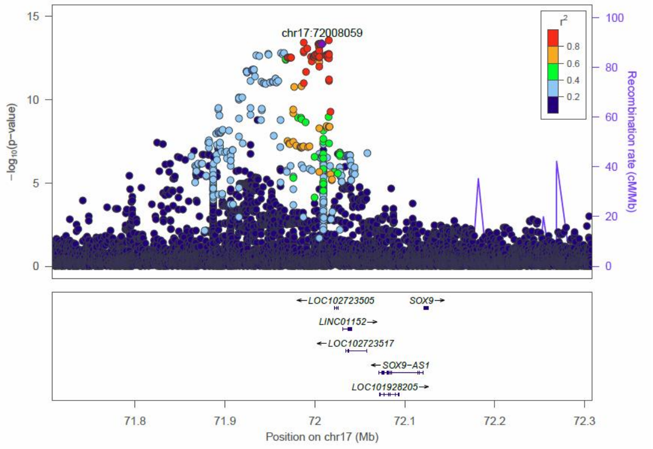

m) Hip total 17q23

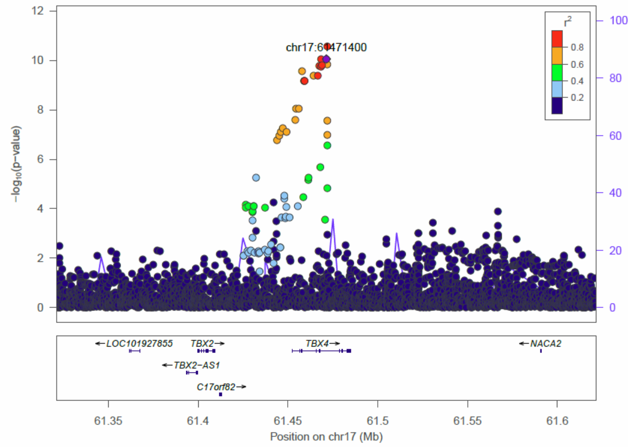

p) Hip intertrochanter area 17q24.3_2

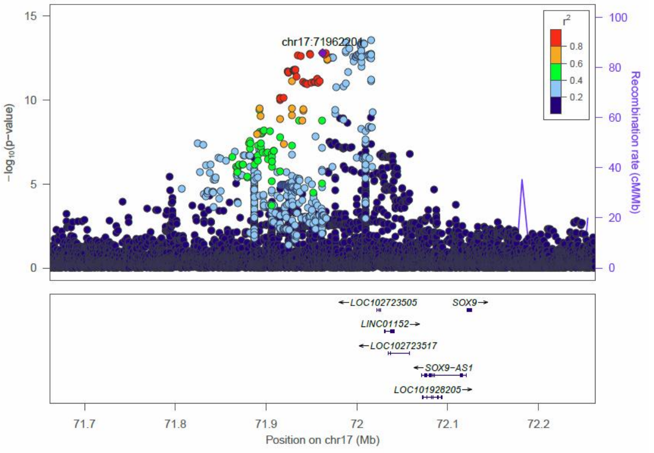

q) Hip intertrochanter area 1p21.1

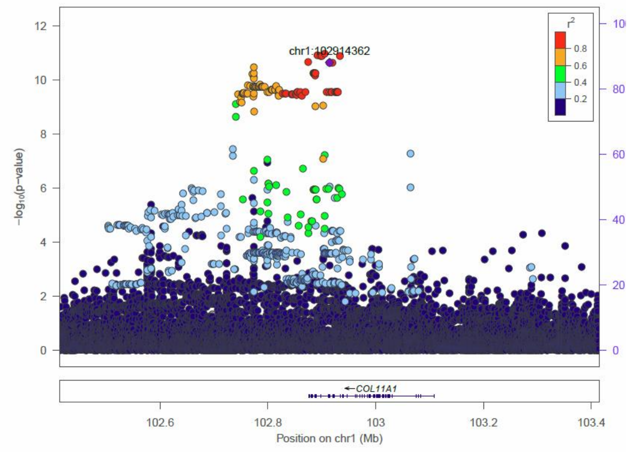

t) Hip trochanter area 1p21

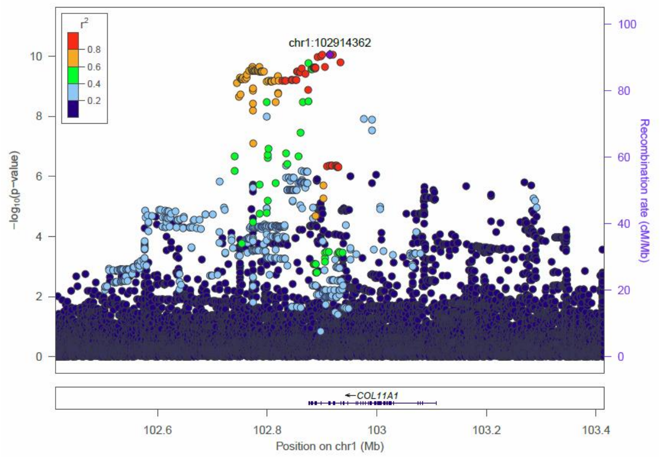

r) Hip intertrochanter area 3p14.3

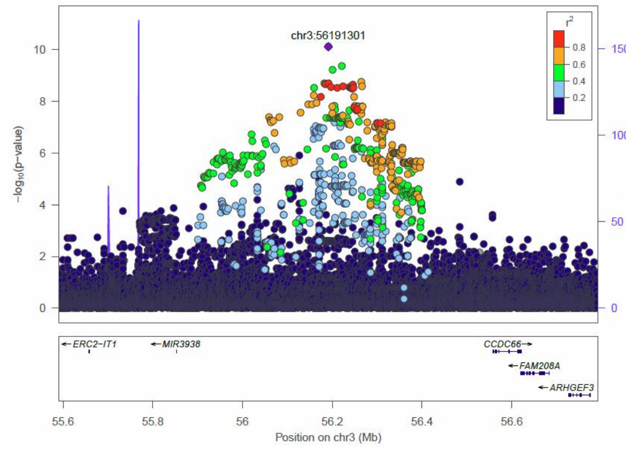

u) Hip trochanter area 12q14.1

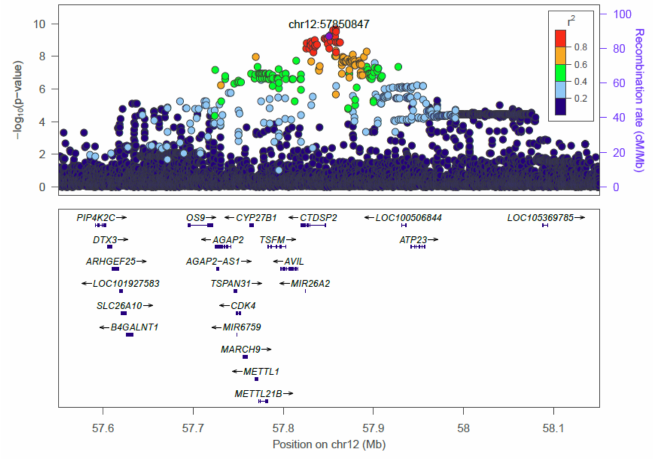

s) Hip trochanter area 20q11.22

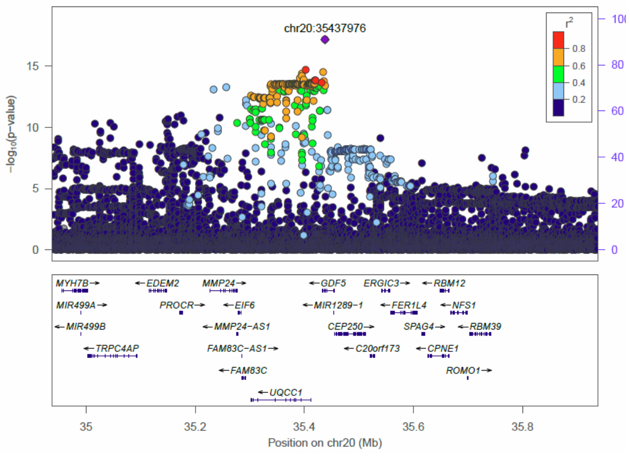

Supplementary Figure 3.   Effect of rs11614913 on mir-196-a2

a) **b)**

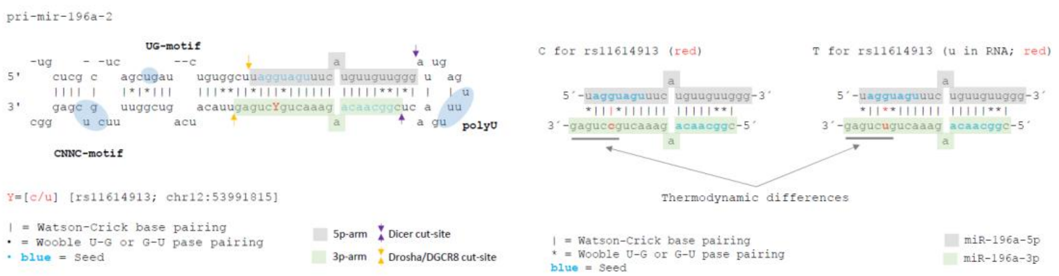

a) The secondary structure of miR-196a2 primary transcript (adapted from mirbase.org) showing the position of the rs11614913 variant with respect to known miR motifs and cut sites used by Drosha (orange arrows) and Dicer (purple arrows). The variant resides on the 3p-arm (the passanger strand), whereas it is the 5p-arm that represents the guiding strand for miR-196a-2 used by Ago for post-transcriptional regulation of target mRNA´s. **b**) The mature miR-196a-2 duplex with C- and T-alleles (rs11614913) shown to the left and right, respectively. The C-allele (left) results in a Watson-Crick bond in the mature miR duplex whereas the T-allele (right), U in RNA, introduces a wobble bond (asterisk signs).

Supplementary Figure 4. Fraction of genes with predicted miR-196a-5p 3´UTR recognition sites that have experimental support

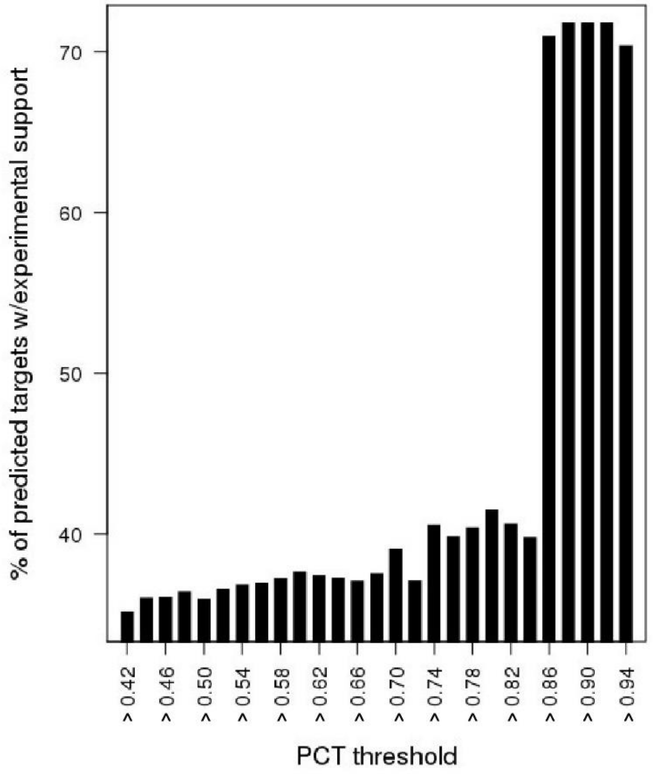

The fraction of genes with *in silico* predicted 3´UTR recognition sites for miR-196a-5p that have experimental support (Tarbase V8) increases for predictions with higher PCT scores. Source data are provided as a Source Data file.

Supplementary Figure 5. The effect of the rs11614913 variant in MIR196A2 on target gene expression in human HEK293 cell line

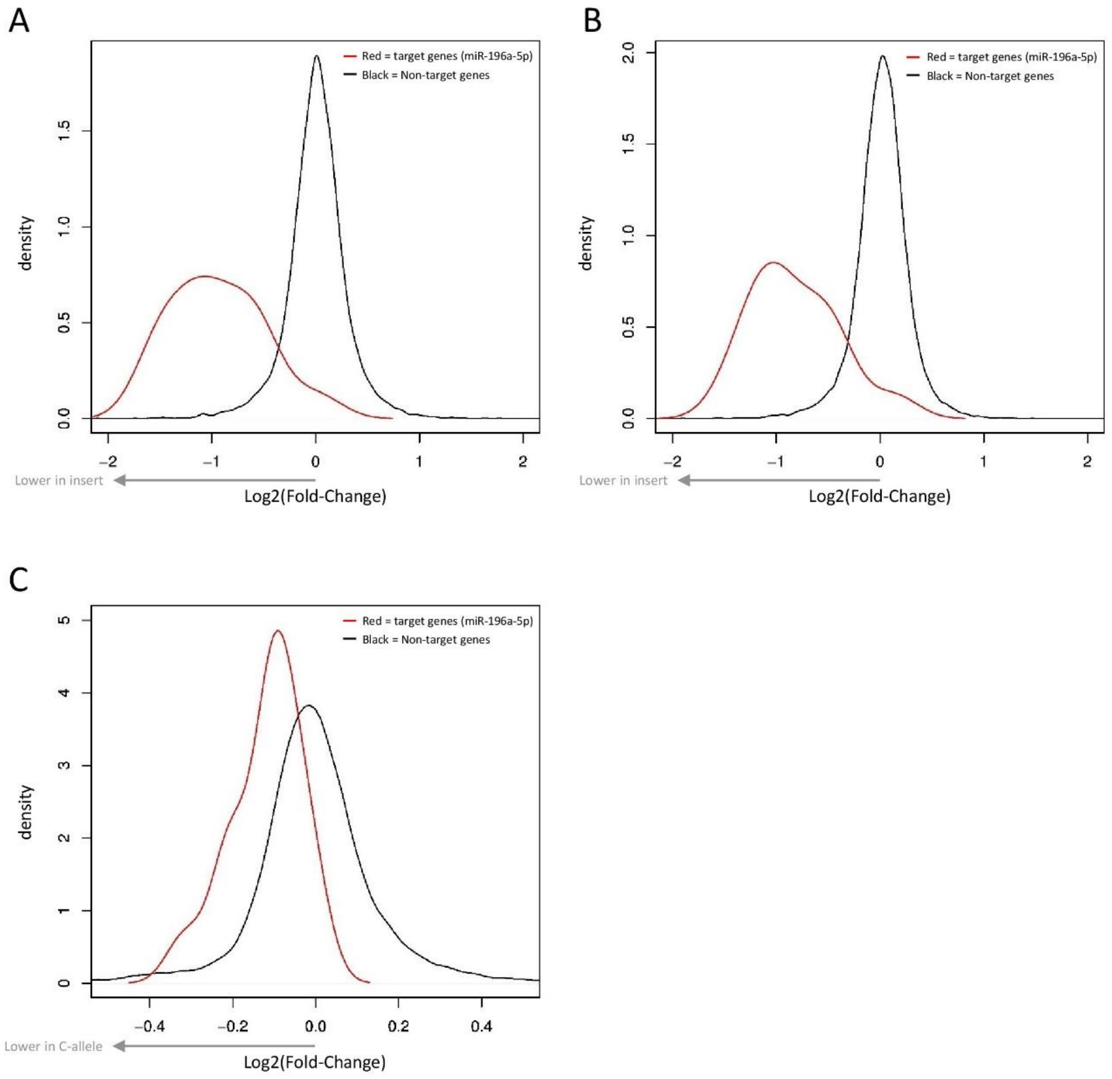

Density plots shown for log2 fold-change values derived from all pair-wise comparisons; A) C-allele compared with empty vector insert; B) T-allele compared with empty vector insert and C) C-allele compared with T-allele where negative log2-fold values reflect downregulation in C-allele. In all instances, the red lines represent density of the fourteen miR-196a-5p target genes whereas the black line represents other non-target genes. The direction of effect for each plot is indicated below the x-axis (grey arrows). Source data are provided as a Source Data file.

Supplementary Figure 6. Downregulation in MIR196A2 insert cells for genes with predicted 3'UTR recognition sites.

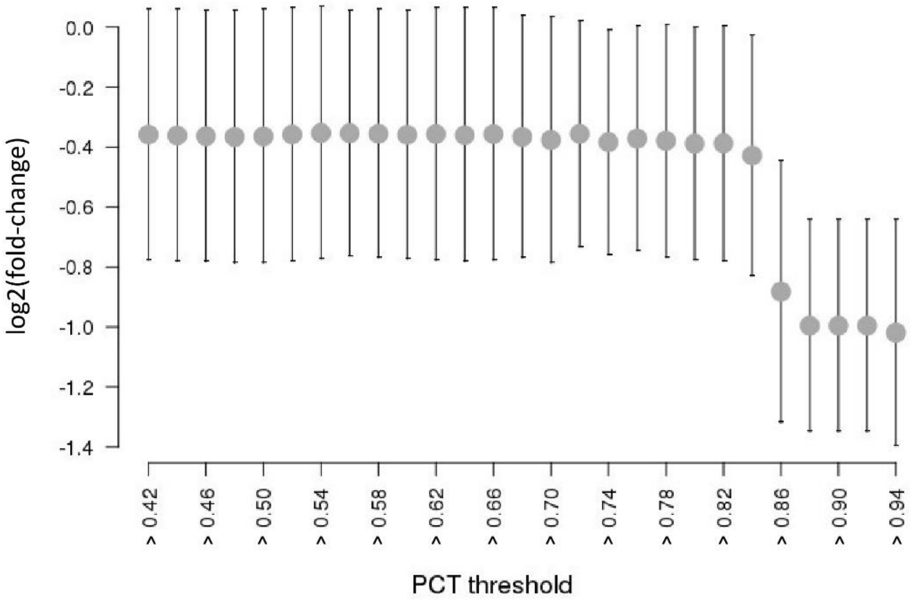

The mean for log2 fold change values derived from MIR196A2 insert versus empty vector controls for genes with *in silico* predicted 3´UTR recognition sites shown as grey dots (y-axis) calculated at incremental steps of thresholding the P_{CT} score (x-axis); the lines represent one standard deviation from the mean. with respect to P_{CT} score. Negative log2 fold change values represents downregulation in MIR196A2 insert cells compared with empty controls. Note the change point for the mean in log2 fold change values at $P_{CT} > 0.86$, highlighting the value of using P_{CT} score for defining a list of high-confidence miR-196a-5p target genes. Source data are provided as a Source Data file.

Supplementary Figure 7. Gene set enrichment for the entire set of genes suppressed by MIR196A insert.

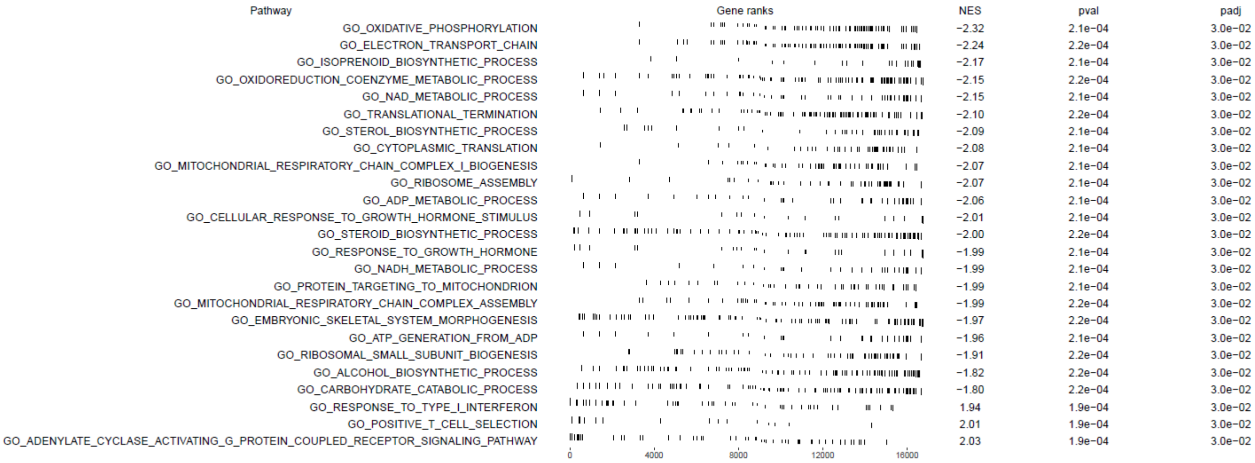

Gene set enrichment for the MIR196A insert versus empty vector control comparison. In this analysis, the entire set of genes tested for differential expression are ranked on the basis of log2 fold change values, and then assessed for whether specific functional gene ontology terms are enriched at the high or low end of the gene rankings. We find significant enrichment (P-adjusted < 0.05) for 31 functional GO terms ordered on the figure from negative to positive NES enrichment values, from top to bottom, respectively. The negative NES values represent enriched GO terms among downregulated genes and *vice versa*. Source data are provided as a Source Data file.

Supplementary Figure 8. Correlation between rs11614913 and rs371683123 at 12q13.13 and expression of the *HOXC8* gene in adipose tissue.

a) **b)**

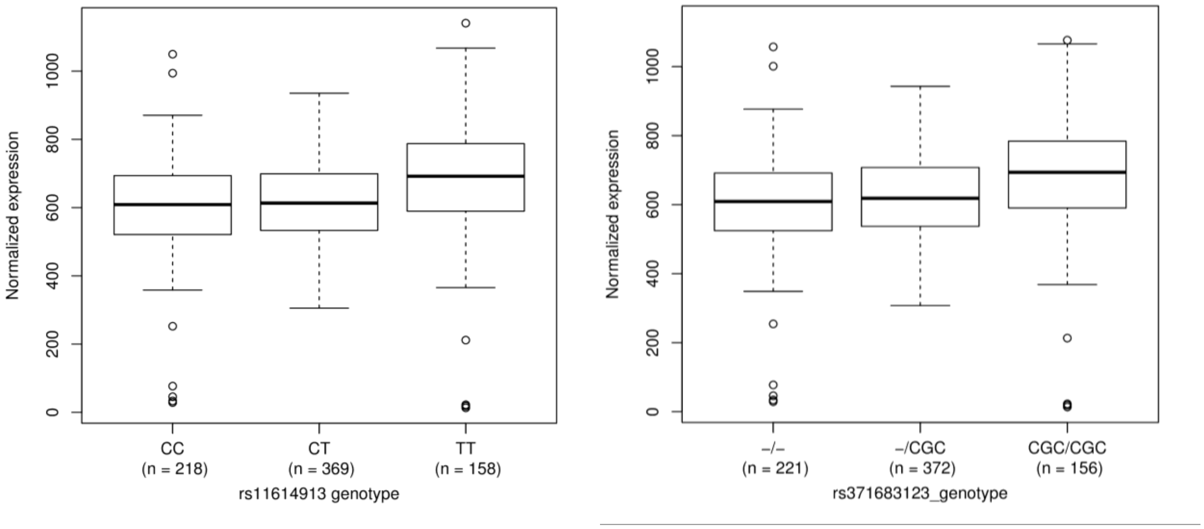

a) Correlation between expression of HOXC8 in adipose tissue and genotypes of a) rs11614913 ($P = 5.9 \times 10^{-9}$, $\beta = 0.31$), and b) rs371683123 ($P = 1.3 \times 10^{-9}$, $\beta = 0.32$). The number of individuals with the respective genotypes are shown below the genotype.

The bottom and top of the box represent first and third quartiles, the line inside the box is the median and whiskers +/- 1.5 interquartile range. The open circles represent outliers that lie beyond the extremes of the whiskers.

Association between variant and gene expression was estimated using a generalized linear regression, assuming additive genetic effect and log-transformed gene expression estimates, adjusting for measurements of sequencing artefacts, demography variables, and hidden covariates. Source data are provided as a Source Data file.

Supplementary Figure 9. Correlation between effects of GIANT height variants on adult height and effects on DXA areas

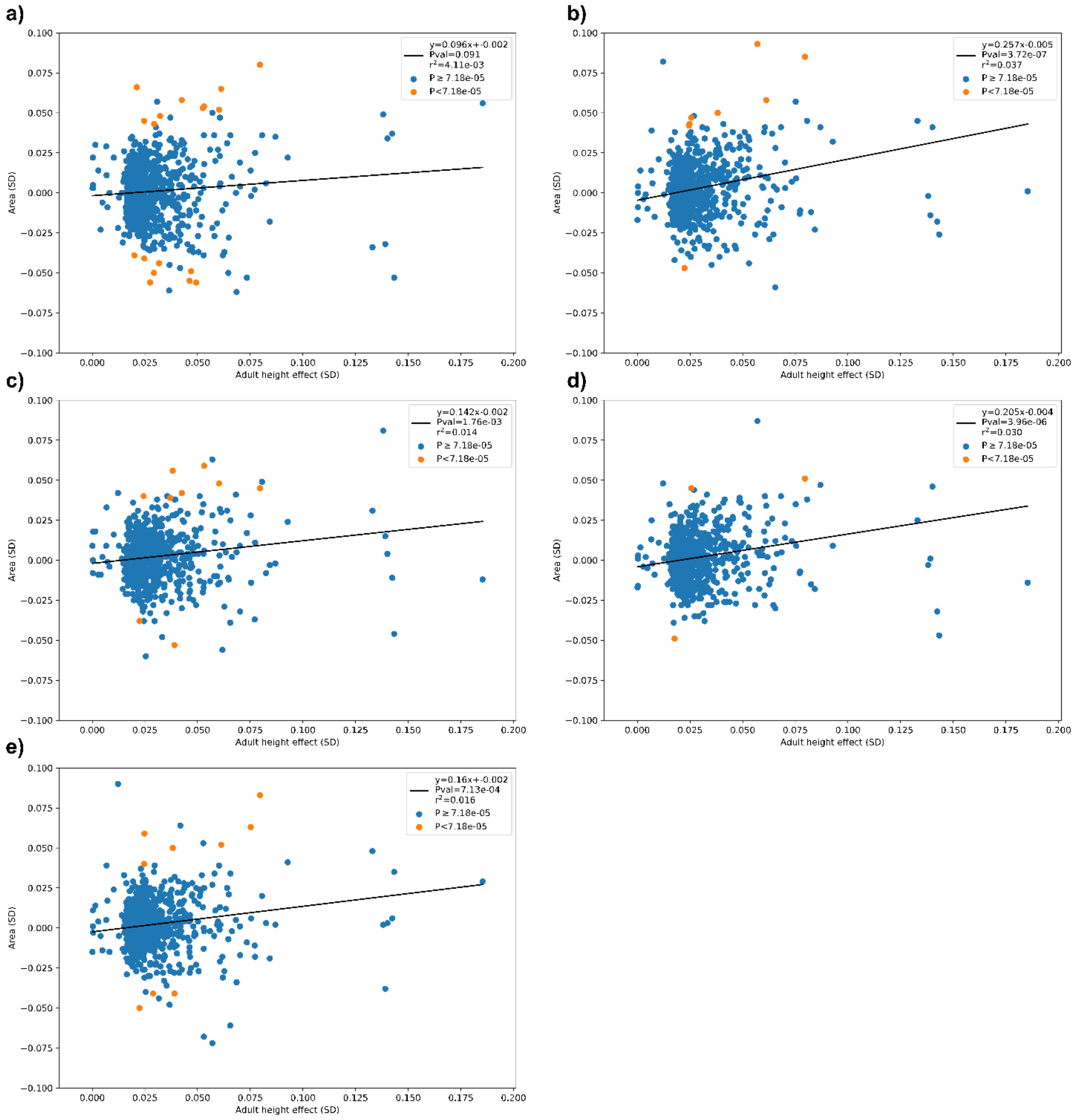

Scatter plot showing correlation between the effects on adult height of the 697 significant variants reported by GIANT [10] (x-axis) and area measures (y-axis) in our data. The variants effect on height is derived from the GIANT dataset excluding the Icelandic data. The allele used for each variant is the one that has a positive effect on adult height. The black solid line represents linear regression weighted by allele frequency AF (AF*(1-AF)). The orange colored points represent significant associations with area measures ($P \leq 7.18 \times 10^{-5}$), accounting for the 697 variants tested. **a**) Spine area (L1-L4), **b**) Total hip area, **c**) Femoral neck area, **d**) Intertrochanteric area, **e**) Trochanter area. Source data are provided as a Source Data file.

Supplementary Figure 10. Correlation between effects of GEFOS BMD variants on BMD and effects on DXA areas

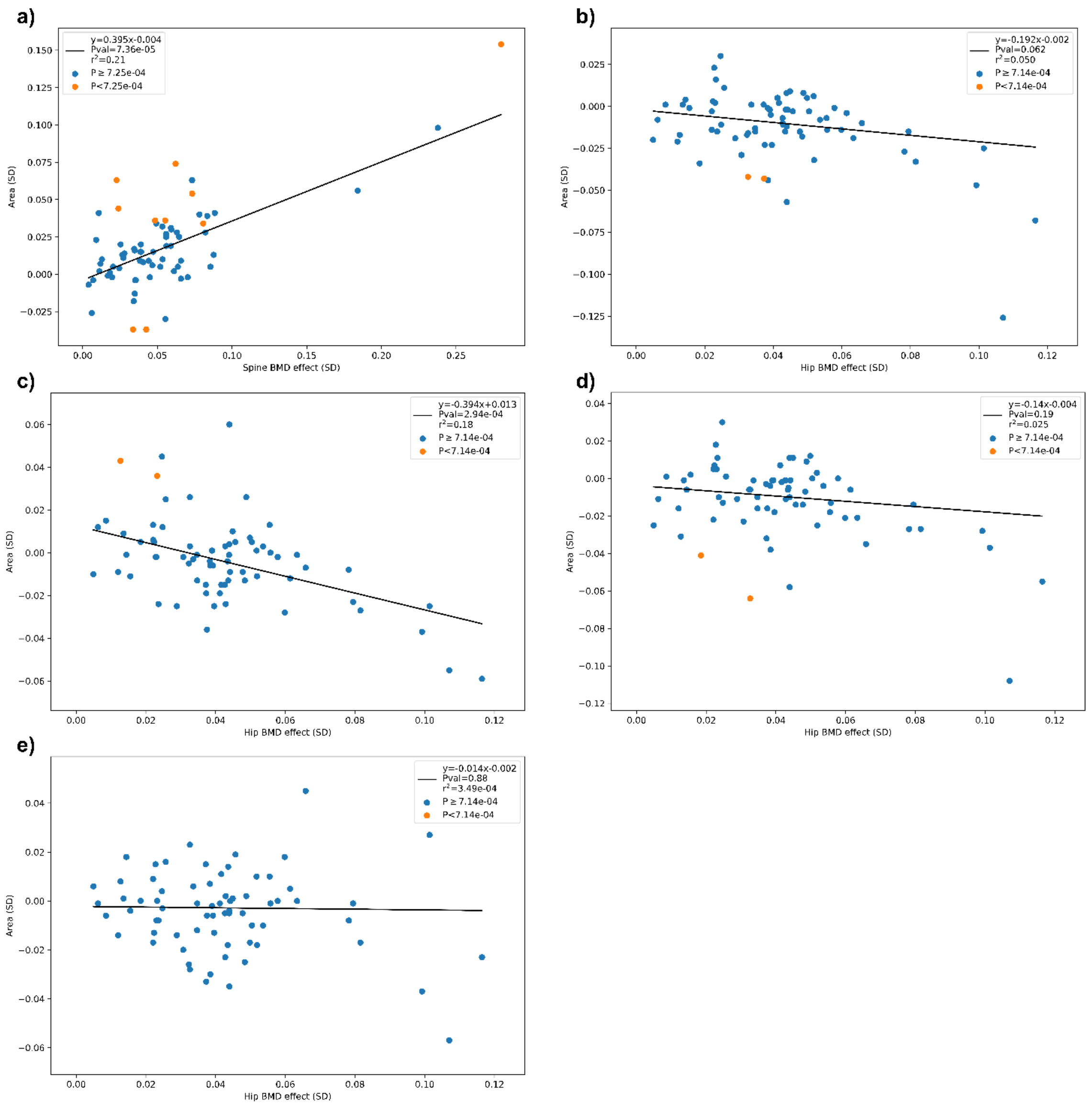

Scatter plot showing correlation between the effects on BMD in the GEFOS dataset [11] (x-axis) between the reported 72 significant variants and area measures (y-axis) in our data. This dataset did not include the Icelandic samples in the meta-analysis. The allele used of each variant is the one that has a positive effect on BMD. The black solid line represents linear regression weighted by allele frequency AF (AF*(1-AF)). The orange colored points represent significant associations with area measures ($P \leq 7.1 \times 10^{-4}$), accounting for the 72 variants tested. **a**) Spine area (L1-L4) and spine BMD, **b**) Total hip area and hip BMD, **c**) Femoral neck area and hip BMD, **d**) Intertrochanteric area and hip BMD, **e**) Trochanter area and hip BMD. Source data are provided as a Source Data file.

Supplementary Figure 11.   Gating strategy to sort GFP positive HEK293T cells

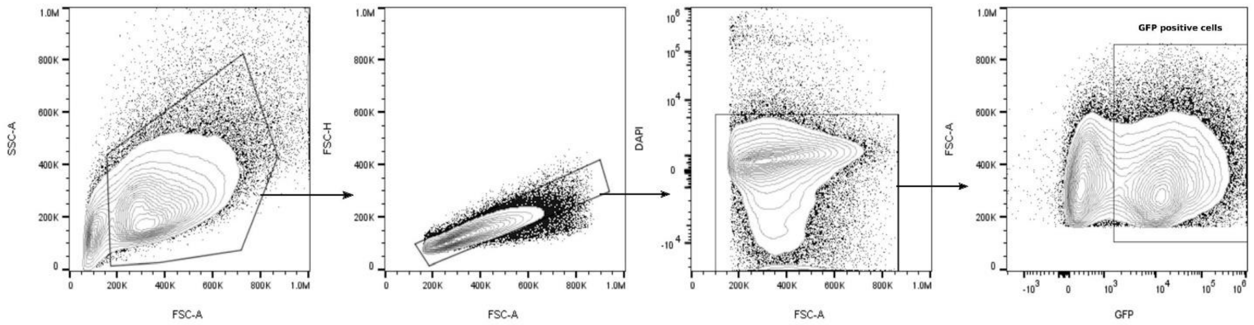

Supplementary Tables

Supplementary Table 1. Conditional analysis between variants at three loci in the Icelandic discovery dataset: a) between two variants at 15q25.2 at the *ADAMTSL3* and *SH3GL3* genes, b) between the two variants at the *SOX9* locus that associate with intertrochanteric area in the Icelandic discovery dataset and two recently published variants that associate with hip shape measures, and c) between the 4q31.21 Icelandic variant and a recently published variant associated with hip shape measure

a) 15q25.2, spine area

				rs2585073		rs8036748	
**rs_name**	**Pos_build38**	**Effect (SD)**	**P**	r^2	P_{adj}	r^2	P_{adj}
rs8036748	chr15:83903084	-0.060	1.3E-10	0.245	1.3E04	–	–
rs2585073	chr15:83597138	0.063	1.3E-10	–	–	0.245	1.2E-4

b) 17q24.3, intertrochanteric area

				rs12601029		rs1159421		rs2158915*		rs2160442*	
**rs_name**	**Pos_build38**	**Effect (SD)**	**P**	r^2	P_{adj}	r^2	P_{adj}	r^2	P_{adj}	r^2	P_{adj}
rs12601029	chr17:72007174	0.074	4.2E-14	–	–	0.371	1.8E-4	0.966	0.094	0.998	0.42
rs1159421	chr17:71962201	-0.069	6.4E-14	0.371	2.8E-4	–	–	0.358	2.3E-4	0.371	2.0E-4
rs2158915*	chr17:72004543	0.074	7.4E-14	0.966	0.18	0.358	2.5E-4	–	–	0.965	0.023
rs2160442*	chr17:72008059	0.075	4.6E-14	0.998	0.47	0.371	1.4E-4	0.965	0.29	–	–

rs2158915 and rs2160442 associate with hip shape measures HSM1 and HSM5, respectively (Baird, et al., JBMR, 2018)

c) 4q31.21, femoral neck area

rs_name	Pos_build38	Effect (SD)	P	rs6537291*		rs12507427	
				r^2	P_{adj}	r^2	P_{adj}
rs12507427	chr4:144646319	0.059	1.2E-10	0.114	3.10E-7	–	–
rs6537291*	chr4:144542922	0.046	6.8E-7	–	–	0.114	3.1E-3

rs6537291* associates with hip shape measure HSM2 (Baird, et al., JBMR, 2018)

Supplementary Table 2. The number of individuals in all participating studies split by phenotype

	Iceland deCODE	Denmark PERF	The Netherlands Rotterdam I,II,III	Australia DOES	UK UKBiobank	UK arcOGEN	Sweden MDC	Hong Kong Mr/Ms OS	Korea AMC	Vietnam VOS	Total
**DXA area measures**											
Spine L1-L4	29,059	4,067	5,208	1,262	4,395			3,755	650	1,940	50,336
Hip total	28,900	1,707	6,205					3,753		1,946	42,511
Femoral neck	28,954	1,707	6,413	1,263				3,753	652	1,946	44,688
Intertrochanteric	28,936	1,707	6,205					3,753		1,943	42,544
Trochanteric	28,944	1,705	6,360					3,753		1,958	42,720
**Hip osteoarthritis**											
cases	5,134	413	657		11,437	3,312	505				21,458
controls	183,824	1,538	8,643		397,218	9,268	876				601,367
**Knee osteoarthritis**											
cases	3,322	460	1,486		19,205	2,421	427				27,321
controls	144,202	1,538	7,437		389,362	9,268	876				552,683
**Hip fractures**											
cases	8,068	193		129	1,788						10,178
controls	296,532	1,538		516	405,154						703,740
**Vertebral fractures**											
cases	1,317	877		200	899						3,293
controls	178,399	1,538		516	407,668						588,121

The number of samples in each sample-set are shown for the respective phenotype. The country of origin of the sample-set is shown and the study acronym (see Methods). deCODE is the deCODE genetics study, PERF is Prospective Epidemiological Risk Factor study, Rotterdam I,II,III is the Rotterdam studies, DOES is Dubbo Osteoporosis Epidemiology Study, arcOGEN is the Arthritis Research UK Osteoarthritis Genetics Study, MDC is the Swedish Malmo Diet and Cancer study, Mr/Ms OS are the Mr and Ms Osteoporosis studies, AMC is Asan Medical Center study, and VOS is the Vietnam Osteoporosis Study.

Supplementary Table 3. Gene function analysis. A) Analysis of gene ontology overrepresentation for the high-confidence miR-196a-5p target genes as carried out in Panther (pantherdb.org); showing results at FDR < 5% ordered according to decreasinging level of overrepresentation ratios. B) The seventeen miR-196a-5p target genes listed out with mean expression in HEK293T (inhouse data) and osteoblasts (Fantom5 data).

A)	GO biological process	Overrepresentation (observed/expected)	P-value	FDR
	anterior/posterior pattern specification (GO:0009952)	25.17	1.68E-05	0.024
	skeletal system development (GO:0001501)	13.56	2.36E-05	0.029
	regulation of transcription by RNA polymerase II (GO:0006357)	4.98	3.85E-06	0.012
	regulation of transcription, DNA-templated (GO:0006355)	4.17	4.77E-06	0.013
	regulation of nucleic acid-templated transcription (GO:1903506)	4.11	5.54E-06	0.013
	regulation of RNA biosynthetic process (GO:2001141)	4.1	5.67E-06	0.011
	regulation of cellular macromolecule biosynthetic process (GO:2000112)	4.06	1.40E-06	0.022
	regulation of macromolecule biosynthetic process (GO:0010556)	3.93	1.97E-06	0.016
	regulation of RNA metabolic process (GO:0051252)	3.85	1.07E-05	0.017
	regulation of cellular biosynthetic process (GO:0031326)	3.81	2.84E-06	0.015
	regulation of biosynthetic process (GO:0009889)	3.74	3.47E-06	0.014
	regulation of nucleobase-containing compound metabolic process (GO:0019219)	3.59	2.16E-05	0.028
	regulation of gene expression (GO:0010468)	3.52	6.72E-06	0.012

B)	HGNC names for miR-196a-5p target genes	Offical full name	Ontology GO:0001501 (skeletal system development)	Osteoblasts; median (min, max)*
	GAN	gigaxonin	No	3.31 (2.66,7,24)
	HMGA2	high mobility group AT-hook 2	Yes	43.46 (31.3, 92,33)

HOXA7	homeobox A7	Yes	3.13 (2.07, 9.1)
HOXA9	homeobox A9	Yes	9.96 (5.35, 12.42)
HOXC8	homeobox C8	Yes	3.31 (0, 7.31)
LCOR	ligand dependent nuclear receptor corepressor	No	4.51 (2.27, 14.61)
LIN28B	lin-28 homolog B	No	0 (0, 0)
NR6A1	nuclear receptor subfamily 6 group A member 1	No	0 (0, 0.18)
NRAS	NRAS proto-oncogene, GTPase	No	31.3 (11.37, 89.01)
PBX1	PBX homeobox 1	Yes	2.03 (0, 12.42)
POTEG	POTE ankyrin domain family member G	No	Unknown
POTEM	POTE ankyrin domain family member D	No	Unknown
PPP1R15B	protein phosphatase 1 regulatory subunit 15B	No	18.19 (13.84, 22.58)
RP1-170O19.20	Uncharacterized protein	Unknown	Unknown
USP15	ubiquitin specific peptidase 15	No	18.36 (4.55, 32.55)
ZBTB26	zinc finger and BTB domain containing 26	No	2.21 (0.66, 3.01)
ZMYND11	zinc finger MYND-type containing 1	No	7.19 (6.02, 13.95)

* Fantom5 TSS dataset; median (min, max) TPM from osteoblasts, sample ID´s: CNhs11385, CNhs11980, CNhs11311, CNhs12036, CNhs12035

Supplementary Table 4. Differential expression of miR-196a-5p target genes in pairwise comparisons for gene expression experiments carried out in cells transfected with MIR196A2 C-allele insert, T-allele insert and empty vector control

A) T-allele versus empty controls*

**Gene**	**target_id**	**Chr**	**Start**	**End**	**Strand**	**log2 FoldChange**	**P-value**	**P-value (adjusted)**
HOXA7	ENSG00000122592.7	chr7	27153716	27156677	-	-1.46	5.43E-29	4.73E-26
NR6A1	ENSG00000148200.16	chr9	124517275	124771277	-	-0.96	1.76E-26	1.18E-23
HMGA2	ENSG00000149948.13	chr12	65824131	65966295	+	-1.26	9.70E-21	3.12E-18
HOXA9	ENSG00000078399.16	chr7	27162435	27165530	-	-1.10	1.15E-19	3.26E-17
PBX1	ENSG00000185630.18	chr1	164555584	164564015	+	-0.70	3.48E-19	9.53E-17
HOXC8	ENSG00000037965.5	chr12	54009106	54012362	+	-1.09	1.02E-17	2.46E-15
LIN28B	ENSG00000187772.7	chr6	104957048	105083332	+	-1.23	1.01E-14	1.54E-12
NRAS	ENSG00000213281.4	chr1	114704469	114716894	-	-0.89	3.95E-12	4.33E-10
ZMYND11	ENSG00000015171.18	chr10	134465	237651	+	-1.00	2.67E-11	2.59E-09
PPP1R15B	ENSG00000158615.8	chr1	204403387	204411791	-	-0.63	9.72E-07	4.22E-05
LCOR	ENSG00000196233.12	chr10	96832260	96964431	+	-0.48	0.0002	0.0041
ZBTB26	ENSG00000171448.8	chr9	122915566	122931500	-	-0.44	0.0002	0.0048
GAN	ENSG00000261609.4	chr16	81314952	81390884	+	-0.40	0.0268	0.1669
USP15	ENSG00000135655.14	chr12	62260338	62416389	+	0.14	0.3645	0.6748

*Negative log2FC reflect lower expression in T-allele

B) C-allele versus empty controls*

**Gene**	**target_id**	**Chr**	**Start**	**End**	**Strand**	**log2 FoldChange**	**P-value**	**P-value (adjusted)**
HMGA2	ENSG00000149948.13	chr12	65824131	65966295	+	-1.47	7.68E-63	2.37E-59
HOXA7	ENSG00000122592.7	chr7	27153716	27156677	-	-1.51	1.83E-59	4.70E-56
NR6A1	ENSG00000148200.16	chr9	124517275	124771277	-	-1.07	1.80E-41	1.47E-38
HOXC8	ENSG00000037965.5	chr12	54009106	54012362	+	-1.31	9.57E-38	5.68E-35
LIN28B	ENSG00000187772.7	chr6	104957048	105083332	+	-1.55	5.44E-35	2.89E-32
PBX1	ENSG00000185630.18	chr1	164555584	164564015	+	-0.80	1.89E-26	4.71E-24
NRAS	ENSG00000213281.4	chr1	114704469	114716894	-	-1.09	2.30E-26	5.63E-24
HOXA9	ENSG00000078399.16	chr7	27162435	27165530	-	-1.12	6.79E-18	7.71E-16
ZMYND11	ENSG00000015171.18	chr10	134465	237651	+	-1.01	2.96E-12	2.00E-10
ZBTB26	ENSG00000171448.8	chr9	122915566	122931500	-	-0.55	1.11E-07	3.69E-06
PPP1R15B	ENSG00000158615.8	chr1	204403387	204411791	-	-0.70	4.15E-07	1.23E-05
LCOR	ENSG00000196233.12	chr10	96832260	96964431	+	-0.57	0.0010	0.0101
GAN	ENSG00000261609.4	chr16	81314952	81390884	+	-0.51	0.0019	0.0175
USP15	ENSG00000135655.14	chr12	62260338	62416389	+	0.02	0.8944	0.9633

*Negative log2FC reflect lower expression in C-allele

C) C-allele versus T-allele*

Gene	target_id	Chr	Start	End	Strand	log2 FoldChange	P-value	P-value (adjusted)
LIN28B	ENSG00000187772.7	chr6	104957048	105083332	+	-0.32	0.0344	0.9974
NRAS	ENSG00000213281.4	chr1	114704469	114716894	-	-0.20	0.0987	0.9974
HMGA2	ENSG00000149948.13	chr12	65824131	65966295	+	-0.20	0.1309	0.9974
HOXC8	ENSG00000037965.5	chr12	54009106	54012362	+	-0.22	0.1358	0.9974
PBX1	ENSG00000185630.18	chr1	164555584	164564015	+	-0.10	0.2065	0.9974
NR6A1	ENSG00000148200.16	chr9	124517275	124771277	-	-0.10	0.3144	0.9974
ZBTB26	ENSG00000171448.8	chr9	122915566	122931500	-	-0.11	0.3189	0.9974
USP15	ENSG00000135655.14	chr12	62260338	62416389	+	-0.12	0.4562	0.9974
GAN	ENSG00000261609.4	chr16	81314952	81390884	+	-0.11	0.5225	0.9974
LCOR	ENSG00000196233.12	chr10	96832260	96964431	+	-0.08	0.5794	0.9974
PPP1R15B	ENSG00000158615.8	chr1	204403387	204411791	-	-0.06	0.6564	0.9974
HOXA7	ENSG00000122592.7	chr7	27153716	27156677	-	-0.05	0.7152	0.9974
HOXA9	ENSG00000078399.16	chr7	27162435	27165530	-	-0.01	0.9243	0.9974
ZMYND11	ENSG00000015171.18	chr10	134465	237651	+	0.00	0.9755	0.9974

*Negative log2FC reflect lower expression in C-allele

Supplementary Table 5. Expression correlation between HOXC8 and rs11614913 and rs371683123 before and after conditioning on the other variant

**SNP**	**Position_build38**	**EA/OA**	**EA Freq.**	**Gene location**	***P* value**	**Effect (s.d.)**	***P* value$_{adj}$**	**Effect (s.d.)$_{adj}$**
rs11614913	chr12:53991815	T/C	47.8	in MIR196A2	5.9E-09	−0.306	0.96	0.008
rs371683123	chr12:54009024	delCGC/CGC	47.7	HOXC8 promoter	1.3E-09	−0.319	0.083	−0.327

We tested the correlation between expression of *HOXC8* in adipose tissue samples from 746 individuals and genotypes of the two variants: rs11614913 in MIR196A2 and rs371683123 in the promoter region of the HOXC8 gene. The two-sided P values and effect, in standard deviations, is shown before and after conditioning on the other variant: rs11614913 before and after conditioning on rs371683123, and rs371683123 before and after conditioning on rs11614913. EA is the effect allele, OA the other allele, EA Freq. is the effect allele frequency.

Supplementary Table 6. Association of DXA area markers with vertebral fractures in all sample-sets combined

								Vertebral fractures (N = 3,293 / 588,121)		
**GWS Phenotype**	**SNP**	**Chr**	**Pos**	**Freq.**	**gene**	**EA**	**OA**	***P* value**	**OR**	***P* het**
Spine	rs11614913	chr12	53991815	47.8	*MIR196A2*	T	C	0.025	1.07	0.055
Spine/Hip_Total/Troch	rs143384	chr20	35437976	37.0	*GDF5*	G	A	0.11	0.96	0.43
Spine	rs10917168	chr1	22187397	28.4	*WNT4*	T	A	0.017	1.08	0.69
Spine	rs143793852	chr18	49083087	43.4	*DYM*	C	CA	0.3	0.97	0.53
Spine	rs8036748	chr15	83903084	46.2	*ADAMTSL3*	A	T	0.78	0.99	0.15
Spine	rs2585073	chr15	83597138	34.9	*SH3GL3*	C	G	0.11	1.05	0.44
Spine	rs9341808	chr6	80243540	47.1	*BCKDHB*	C	A	0.48	0.98	0.043
Spine	rs72979233	chr11	74644478	25.7	*CHRDL2*	G	A	0.74	1.01	0.11
Hip_Total/Inter/Troch	rs3753841	chr1	1.03E+08	39.1	*COL11A1*	G	A	0.78	0.99	0.96
Hip_Total/Inter/Troch	rs9830173	chr3	56191301	38.4	*ERC2*	C	G	0.87	1	0.73
Hip_Total	rs1507462	chr18	75979535	30.4	.	A	G	0.24	1.04	0.86
Hip_Total	rs72834687	chr17	61471400	24.2	*TBX4*	A	G	0.72	0.99	0.56
Hip_FN	rs12507427	chr4	1.45E+08	43.1	*HHIP*	A	T	0.66	0.99	0.82
Hip_Inter	rs12601029	chr17	72007174	33.5	*SOX9*	A	G	0.79	0.99	0.75
Hip_Inter	rs1159421	chr17	71962201	44.7	*SOX9*	T	C	0.99	1	0.85
Hip_Troch	rs10783854	chr12	57850847	35.6	*CTDSP2*	T	C	0.41	1.02	0.23

Results are shown for all sample-sets combined. Phenotype refers to the DXA area phenotypes that were GWS in the discovery analyses. Troch refers to trochanter, Inter to intertrochanter, and FN to femoral neck. EA designate the effect allele and OA the other allele. N designates the number of individuals in the analyses; cases / controls. P het is the heterogeneity P value.

Supplementary Table 7. Association of height markers and BMD markers with DXA area measures in Iceland

rsName	Chr	Pos_build38	EAF	EA	OA	*P* value	Effect	prior_Effect	DXA area phenotype	Prior_phenotype	Gene	Impact
rs1014987	chr1	22172331	0.273	G	C	2.1E-10	0.066	0.021	Spine area	Adult height	.	intergenic
rs143384	chr20	35437976	0.370	G	A	9.9E-17	0.080	0.080	Spine area	Adult height	GDF5	5'UTR
rs1884897	chr20	6632185	0.364	A	G	5.8E-07	-0.049	0.047	Spine area	Adult height	.	intergenic
rs2072153	chr17	49312652	0.317	C	G	4.2E-05	-0.041	0.025	Spine area	Adult height	ZNF652	intron
rs2257011	chr15	83597393	0.480	T	G	5.7E-09	-0.055	0.046	Spine area	Adult height	SH3GL3	intron
rs2425163	chr20	35844748	0.158	G	A	4.3E-07	0.065	0.061	Spine area	Adult height	PHF20	intron
rs3118905	chr13	50531198	0.760	G	A	2.3E-06	0.052	0.060	Spine area	Adult height	.	intergenic
rs3811958	chr5	32771937	0.254	G	A	2.2E-07	-0.056	0.028	Spine area	Adult height	NPR3	intron
rs3825199	chr12	93583178	0.244	G	A	1.4E-06	0.053	0.053	Spine area	Adult height	.	intergenic
rs4834927	chr4	13192467	0.348	G	A	6.8E-05	-0.039	0.020	Spine area	Adult height	.	intergenic
rs4896582	chr6	142382740	0.736	G	A	5.8E-07	0.054	0.053	Spine area	Adult height	ADGRG6	intron
rs648831	chr6	80246491	0.482	T	C	3.6E-07	0.048	0.032	Spine area	Adult height	BCKDHB	intron
rs7043114	chr9	92625701	0.427	C	T	4.9E-06	0.043	0.029	Spine area	Adult height	IPPK	intron
rs7162542	chr15	83845538	0.507	G	C	2.7E-09	-0.056	0.049	Spine area	Adult height	ADAMTSL3	intron
rs7517682	chr1	103054033	0.449	G	A	1.5E-06	0.045	0.025	Spine area	Adult height	COL11A1	intron
rs7567288	chr2	133677253	0.207	C	T	1.7E-05	-0.050	0.029	Spine area	Adult height	.	intergenic
rs9967417	chr18	49433130	0.433	G	C	1.1E-09	0.058	0.043	Spine area	Adult height	DYM	intron
rs9993613	chr4	72610297	0.508	T	G	2.5E-06	-0.044	0.032	Spine area	Adult height	.	intergenic
rs10748128	chr12	69433878	0.385	T	G	3.0E-07	0.050	0.038	Total hip area	Adult height	.	intergenic
rs10877030	chr12	57862931	0.648	T	G	1.4E-05	0.043	0.025	Total hip area	Adult height	.	intergenic
rs2425163	chr20	35844748	0.158	G	A	8.7E-06	0.058	0.061	Total hip area	Adult height	PHF20	intron
rs6879260	chr5	180304014	0.589	C	T	1.0E-06	0.047	0.026	Total hip area	Adult height	GFPT2	intron

rs7517682	chr1	103054033	0.449	G	A	9.7E-06	0.042	0.025	Total hip area	Adult height	COL11A1	intron
rs763318	chr4	12961950	0.541	G	A	8.2E-07	-0.047	0.022	Total hip area	Adult height	.	intergenic
rs9456307	chr6	158508410	0.954	T	A	4.5E-05	0.093	0.057	Total hip area	Adult height	TULP4	3'UTR
rs10748128	chr12	69433878	0.385	T	G	2.4E-09	0.056	0.038	Femoral neck area	Adult height	.	intergenic
rs10859567	chr12	93733149	0.543	T	G	1.9E-05	0.039	0.037	Femoral neck area	Adult height	CRADD	intron
rs11049611	chr12	28447311	0.694	C	T	1.4E-07	-0.053	0.039	Femoral neck area	Adult height	CCDC91	intron
rs143384	chr20	35437976	0.370	G	A	2.5E-06	0.045	0.080	Femoral neck area	Adult height	GDF5	5'UTR
rs2338115	chr17	38773325	0.523	T	C	1.6E-05	0.040	0.024	Femoral neck area	Adult height	PIP4K2B	intron
rs3118905	chr13	50531198	0.760	G	A	8.8E-06	0.048	0.060	Femoral neck area	Adult height	.	intergenic
rs4240326	chr4	144918112	0.476	A	G	6.3E-06	0.042	0.043	Femoral neck area	Adult height	.	intergenic
rs6845999	chr4	144644674	0.431	T	C	1.8E-10	0.059	0.053	Femoral neck area	Adult height	HHIP	upstream
rs763318	chr4	12961950	0.541	G	A	3.1E-05	-0.038	0.022	Femoral neck area	Adult height	.	intergenic
rs10083886	chr17	71927214	0.210	T	C	1.6E-05	-0.049	0.018	Intertrochanter area	Adult height	.	intergenic
rs143384	chr20	35437976	0.370	G	A	1.1E-07	0.051	0.080	Intertrochanter area	Adult height	GDF5	5'UTR
rs6879260	chr5	180304014	0.589	C	T	1.8E-06	0.045	0.026	Intertrochanter area	Adult height	GFPT2	intron
rs10748128	chr12	69433878	0.385	T	G	1.9E-07	0.050	0.038	Trochanter area	Adult height	.	intergenic
rs10877030	chr12	57862931	0.648	T	G	1.4E-09	0.059	0.025	Trochanter area	Adult height	.	intergenic
rs11049611	chr12	28447311	0.694	C	T	6.2E-05	-0.041	0.039	Trochanter area	Adult height	CCDC91	intron

rs143384	chr20	35437976	0.370	G	A	6.7E-18	0.083	0.080	Trochanter area	Adult height	GDF5	5'UTR
rs1582931	chr5	123321505	0.518	G	A	1.1E-05	-0.041	0.029	Trochanter area	Adult height	.	intergenic
rs2425163	chr20	35844748	0.158	G	A	4.5E-05	0.052	0.061	Trochanter area	Adult height	PHF20	intron
rs7517682	chr1	103054033	0.449	G	A	1.7E-05	0.040	0.025	Trochanter area	Adult height	COL11A1	intron
rs763318	chr4	12961950	0.541	G	A	7.1E-08	-0.050	0.022	Trochanter area	Adult height	.	intergenic
rs7692995	chr4	17935011	0.897	T	C	3.8E-05	0.063	0.075	Trochanter area	Adult height	LCORL	intron
rs115242848	chr2	118750031	0.012	T	C	3.4E-04	0.154	0.280	Spine area	Spine BMD	EN1	intergenic
rs11755164	chr6	44671447	0.643	C	T	9.3E-06	0.044	0.024	Spine area	Spine BMD	.	intergenic
rs2062377	chr8	118995181	0.451	T	A	2.9E-04	0.034	0.081	Spine area	Spine BMD	.	intergenic
rs227584	chr17	44148179	0.273	C	A	5.6E-04	-0.037	0.043	Spine area	Spine BMD	C17orf53	p.Thr126Pro
rs3801387	chr7	121334711	0.221	G	A	1.5E-06	0.054	0.073	Spine area	Spine BMD	WNT16	intron
rs4233949	chr2	54432570	0.354	C	G	2.8E-04	0.036	0.055	Spine area	Spine BMD	.	intergenic
rs577721086	chr6	127118902	0.073	C	T	5.5E-04	0.063	0.023	Spine area	Spine BMD	RSPO3	upstream
rs6532023	chr4	87852697	0.295	T	G	4.6E-04	0.036	0.048	Spine area	Spine BMD	.	intergenic
rs736825	chr12	54023792	0.603	C	G	1.0E-14	0.074	0.062	Spine area	Spine BMD	HOXC6	upstream
rs7521902	chr1	22164231	0.720	C	A	4.3E-04	-0.037	0.034	Spine area	Spine BMD	.	intergenic
rs11755164	chr6	44671447	0.643	C	T	6.7E-06	0.043	0.013	Total hip area	Hip BMD	.	intergenic
rs13245690	chr7	121145010	0.624	A	G	1.2E-04	0.036	0.023	Total hip area	Hip BMD	CPED1	intron
rs163879	chr11	30930127	0.286	C	T	8.2E-05	-0.041	0.018	Total hip area	Hip BMD	DCDC5	intron
rs7217932	chr17	71952875	0.440	A	G	8.7E-12	-0.064	0.033	Total hip area	Hip BMD	.	intergenic
rs7217932	chr17	71952875	0.440	A	G	1.2E-05	-0.042	0.033	Total hip area	Hip BMD	.	intergenic
rs7521902	chr1	22164231	0.720	C	A	4.4E-05	-0.043	0.037	Total hip area	Hip BMD	.	intergenic

Results are shown for markers that are significant after correcting for multiple testing (Bonferroni P < 7.2E-5 for height, and < 0.00071 for BMD). The allele used of each variant is the one that has a positive effect on adult height or positive effect on BMD. Chromosomal location is according to build38. Effect refers to the area phenotype. Prior effect refers to the effect on height or on BMD. Prior phenotype is either height or weight. EA is the effect allele, EAF the effect allele frequency, and OA the other allele.

Supplementary Table 8. Mendelian Randomization

Outcome	Exposure	Method	Estimate	Std Error	95% CI	P-value
FN BMD	Total hip area	Simple median	0.164	0.057	0.053,0.275	0.004
		Weighted median	0.127	0.051	0.028,0.226	0.012
		IVW	0.178	0.045	0.091,0.266	0.000
		MR-Egger	0.059	0.105	-0.148,0.265	0.577
		(intercept)	0.006	0.005	-0.003,0.015	0.210
	Inter area	Simple median	0.184	0.054	0.078,0.290	0.001
		Weighted median	0.112	0.049	0.015,0.209	0.023
		IVW	0.179	0.046	0.089,0.270	0.000
		MR-Egger	0.006	0.107	-0.204,0.216	0.955
		(intercept)	0.008	0.005	-0.001,0.017	0.074
	Trochanter area	Simple median	0.018	0.054	-0.088,0.124	0.740
		Weighted median	0.000	0.050	-0.097,0.097	1,000
		IVW	0.031	0.039	-0.045,0.108	0.424
		MR-Egger	0.035	0.093	-0.147,0.217	0.707
		(intercept)	0.000	0.004	-0.008,0.008	0.965
	Femoral neck area	Simple median	0.029	0.053	-0.076,0.133	0.589
		Weighted median	0.084	0.049	-0.012,0.181	0.088
		IVW	0.058	0.046	-0.033,0.148	0.212
		MR-Egger	0.249	0.107	0.040,0.459	0.019
		(intercept)	-0.009	0.005	-0.018,0.000	0.047
LS BMD	Lumbar spine area	Simple median	-0.337	0.049	-0.433,-0.241	0.000
		Weighted median	-0.324	0.044	-0.411,-0.237	0.000
		IVW	-0.234	0.052	-0.337,-0.132	0.000
		MR-Egger	-0.307	0.107	-0.518,-0.097	0.004
		(intercept)	0.004	0.005	-0.006,0.015	0.435
Height	Total hip area	Simple median	-0.043	0.025	-0.093,0.006	0.086
		Weighted median	-0.032	0.025	-0.082,0.017	0.200

	IVW	-0.037	0.021	-0.078,0.005	0.085
	MR-Egger	-0.065	0.052	-0.167,0.037	0.210
	(intercept)	0.001	0.002	-0.002,0.004	0.549
Inter area	Simple median	-0.021	0.024	-0.069,0.026	0.380
	Weighted median	0.000	0.024	-0.047,0.047	1,000
	IVW	-0.020	0.019	-0.057,0.016	0.276
	MR-Egger	-0.047	0.045	-0.136,0.041	0.296
	(intercept)	0.001	0.001	-0.002,0.004	0.512
Trochanter area	Simple median	-0.045	0.024	-0.091,0.002	0.060
	Weighted median	-0.030	0.024	-0.077,0.018	0.225
	IVW	-0.044	0.020	-0.083,-0.006	0.024
	MR-Egger	-0.052	0.048	-0.146,0.042	0.279
	(intercept)	0.000	0.001	-0.003,0.003	0.863
Femoral neck area	Simple median	0.022	0.023	-0.024,0.068	0.353
	Weighted median	0.000	0.024	-0.047,0.047	1,000
	IVW	0.004	0.019	-0.033,0.041	0.838
	MR-Egger	-0.012	0.046	-0.102,0.079	0.803
	(intercept)	0.000	0.001	-0.002,0.003	0.714
Lumbar spine area	Simple median	-0.057	0.024	-0.105,-0.009	0.019
	Weighted median	-0.078	0.025	-0.127,-0.028	0.002
	IVW	-0.069	0.023	-0.113,-0.025	0.002
	MR-Egger	-0.102	0.055	-0.209,0.006	0.064
	(intercept)	0.001	0.002	-0.002,0.004	0.510

Causal relationships between DXA bone area and height and BMD were estimated with a two sample Mendelian Randomization, using GIANT and GEFOS effect estimates for height and BMD respectively, as instrumental variables. The Mendelian randomization was performed with the MendelianRandomization package in R[12]. FN is femoral neck, LS is lumbar spine, and Inter refers to intertrochanter.

Supplementary Table 9. Genetic correlations between DXA area, DXA BMD, hip or knee osteoarthritis and hip fractures

						Combined		
	Iceland	UK Biobank	r_g	se	P value	r_g	95% CI	P value
**LS area vs. Other**	LS area	LS BMD	0.35	0.10	9.0E-04	0.30	0.13, 0.46	4.8E-04
	LS BMD	LS area	0.20	0.15	0.17			
	LS area	FN BMD	0.18	0.12	0.13	0.26	0.064, 0.45	9.1E-03
	FN BMD	LS area	0.41	0.17	0.016			
	LS area	Inter BMD	0.20	0.11	0.063	0.22	0.054, 0.39	9.8E-03
	Inter BMD	LS area	0.26	0.14	0.069			
	LS area	Troch BMD	0.16	0.10	0.11	0.23	0.063, 0.39	6.8E-03
	Troch BMD	LS area	0.37	0.15	0.014			
	LS area	Height	0.056	0.038	0.15	0.064	-0.008, 0.14	0.082
	Height	LS area	0.16	0.13	0.22			
	LS area	Hip OA	0.076	0.077	0.32	0.043	-0.10, 0.19	0.55
	Hip OA	LS area	-0.28	0.24	0.25			
	LS area	Knee OA	-0.040	0.059	0.50	-0.034	-0.15, 0.078	0.55
	Knee OA	LS area	0.089	0.26	0.74			
	LS area	Hip fracture	0.27	0.14	0.064	0.22	-0.045, 0.48	0.11
	Hip fracture	LS area	-0.094	0.36	0.80			
**BMD vs BMD**	LS BMD	FN BMD	0.67	0.14	8.9E-07	0.67	0.49, 0.85	1.4E-13
	FN BMD	LS BMD	0.68	0.12	3.1E-08			
	LS BMD	Inter BMD	0.68	0.12	4.6E-08	0.68	0.52, 0.85	6.9E-16
	Inter BMD	LS BMD	0.68	0.12	2.9E-09			
	LS BMD	Troch BMD	0.60	0.13	7.8E-06	0.69	0.52, 0.87	1.9E-14
	Troch BMD	LS BMD	0.77	0.12	3.4E-10			
	FN BMD	Inter BMD	0.98	0.15	1.7E-10	0.97	0.77, 1.18	6.4E-20
	Inter BMD	FN BMD	0.97	0.15	6.4E-11			
	FN BMD	Troch BMD	0.92	0.17	1.2E-07	0.95	0.73, 1.17	4.0E-17
	Troch BMD	FN BMD	0.96	0.15	6.3E-11			

	Troch BMD	Inter BMD	0.98	0.14	1.4E-11	0.97	0.75, 1.18	4.3E-18
	Inter BMD	Troch BMD	0.95	0.17	5.6E-08			
**Hip area vs. Other**	Hip total area	LS area	0.47	0.15	2.4E-03			
	Hip total area	Height	0.12	0.037	1.7E-03			
	Hip total area	Hip OA	0.18	0.073	0.013			
	Hip total area	Knee OA	-0.010	0.057	0.86			
	Hip total area	Hip fracture	0.24	0.14	0.085			
	Hip FN area	LS area	0.43	0.21	0.035			
	Hip FN area	Hip FN BMD	-0.15	0.16	0.36			
	Hip FN area	Height	0.14	0.045	2.7E-03			
	Hip FN area	Hip OA	0.11	0.086	0.19			
	Hip FN area	Knee OA	-0.026	0.075	0.73			
	Hip FN area	Hip fracture	0.76	0.28	6.6E-03			
	Inter area	LS area	0.26	0.15	0.097			
	Inter area	Inter BMD	-0.43	0.14	1.8E-03			
	Inter area	Height	0.093	0.036	9.7E-03			
	Inter area	Hip OA	0.18	0.074	0.019			
	Inter area	Knee OA	-0.020	0.056	0.72			
	Inter area	Hip fracture	0.15	0.13	0.25			
	Troch area	LS area	0.64	0.20	1.6E-03			
	Troch area	Troch BMD	0.031	0.12	0.80			
	Troch area	Height	0.089	0.039	0.023			
	Troch area	Hip OA	0.12	0.079	0.14			
	Troch area	Knee OA	0.021	0.063	0.74			
	Troch area	Hip fracture	0.14	0.15	0.33			
**Hip BMD vs. hip fractures**	Hip fracture	FN BMD	-0.59	0.24	0.014	-0.57	-0.86, -0.28	9.8E-05
	FN BMD	Hip fracture	-0.56	0.19	2.5E-03			
	Hip fracture	Inter BMD	-0.63	0.22	3.9E-03	-0.52	-0.79, -0.25	1.8E-04
	Inter BMD	Hip fracture	-0.45	0.18	0.013			
	Hip fracture	Troch BMD	-0.64	0.23	5.5E-03	-0.44	-0.69, -0.18	7.4E-04
	Troch BMD	Hip fracture	-0.35	0.16	0.029			

We estimated the genetic correlation between the traits indicated using cross-trait LD score regression on the GWAS summary statistics from Iceland and UK Biobank (Methods). rg is the genetic correlation coefficient, se is standard error, and CI confidence interval. LS is lumbar spine, FN is Femoral neck, Inter is intertrochanteric, Troch is trochanteric, OA is osteoarthritis. Hip area measures and total hip BMD are not available in the UK Biobank dataset.

Supplementary Table 10. Contribution of DXA area, BMC and BMD to the risk of hip fractures, full model

**Phenotype**	**OR**	**95% CI**	***P* value**
LS_Area	0.97	0.95, 0.99	0.0063
LS_BMC	1.06	1.01, 1.11	0.027
LS_BMD	0.97	0.94, 1.01	0.16
Hip_Inter_Area	1.10	1.07, 1.13	2.9E-14
Hip_FN_Area	1.01	1.00, 1.01	4.9E-05
Hip_Troch_Area	1.04	1.03, 1.05	3.0E-19
Hip_BMC	0.83	0.79, 0.87	5.8E-14
Hip_FN_BMD	1.01	1.00, 1.02	0.013
Hip_Inter_BMD	1.09	1.06, 1.12	2.8E-08
Hip_Troch_BMD	1.04	1.02, 1.05	2.2E-08

We performed a multivariable logistic regression for hip fractures, using age, height and sex adjusted measurements of DXA area, BMD and BMC of lumbar spine, as well as area and BMD of femoral neck, intertrochanteric and trochanteric and BMC of the total hip as explanatory variables. Each of these phenotypes showed independent association with hip fractures, adjusting for all the other phenotypes, according to likelihood ratio test (see model output below). LS is lumbar spine, Inter is intertrochanteric, Troch in trochanter, FN is femoral neck, BMC is bone mineral content, BMD is bone mineral density, CI is confidence interval, OR is odds ratio.

*** model output.
Single term deletions
Model: hip_fractures ~ LS_area + hip_inter_area + hip_fn_area + hip_troch_area + LS_bmc + LS_bmd + hip_bmc + hip_fn_bmd + hip_inter_bmd + hip_troch_bmd

Supplementary References:

1. Gu, S. *et al.* Thermodynamic stability of small hairpin RNAs highly influences the loading process of different mammalian Argonautes. *Proceedings of the National Academy of Sciences of the United States of America* **108**, 9208-9213 (2011).
2. Acevedo, R., Orench-Rivera, N., Quarles, K.A. & Showalter, S.A. Helical Defects in MicroRNA Influence Protein Binding by TAR RNA Binding Protein. *PLoS ONE* **10**, e0116749 (2015).
3. Connerty, P., Ahadi, A. & Hutvagner, G. RNA Binding Proteins in the miRNA Pathway. *International Journal of Molecular Sciences* **17**, 31 (2016).
4. Karabegović, I. *et al.* Genetic Polymorphism of miR-196a-2 is Associated with Bone Mineral Density (BMD). *International Journal of Molecular Sciences* **18**, 2529 (2017).
5. Bray, N.L., Pimentel, H., Melsted, P. & Pachter, L. Near-optimal probabilistic RNA-seq quantification. *Nature Biotechnology* **34**, 525 (2016).
6. Love, M.I., Huber, W. & Anders, S. Moderated estimation of fold change and dispersion for RNA-seq data with DESeq2. *Genome Biology* **15**, 550 (2014).
7. Karagkouni, D. *et al.* DIANA-TarBase v8: a decade-long collection of experimentally supported miRNA–gene interactions. *Nucleic Acids Research* **46**, D239-D245 (2018).
8. Liberzon, A. *et al.* The Molecular Signatures Database Hallmark Gene Set Collection. *Cell Systems* **1**, 417-425 (2015).
9. Pruim, R.J. *et al.* LocusZoom: regional visualization of genome-wide association scan results. *Bioinformatics* **26**, 2336-2337 (2010).
10. Wood, A.R. *et al.* Defining the role of common variation in the genomic and biological architecture of adult human height. *Nat Genet* **46**, 1173-1186 (2014).
11. Zheng, H.-F. *et al.* Whole-genome sequencing identifies EN1 as a determinant of bone density and fracture. *Nature* **526**, 112-117 (2015).
12. Bowden, J., Davey Smith, G. & Burgess, S. Mendelian randomization with invalid instruments: effect estimation and bias detection through Egger regression. *International Journal of Epidemiology* **44**, 512-525 (2015).
