# Supplementary material for: GWAS of bone size yields twelve loci that also affect height, BMD, osteoarthritis or fractures
Source: Nat Commun. 2019 May 3;10:2054. doi: 10.1038/s41467-019-09860-0 (PMC6499783; doi:10.1038/s41467-019-09860-0)
Supplement: Supplementary file 2 — Description of Additional Supplementary Files [file 41467_2019_9860_MOESM2_ESM.docx]

**Description of Supplementary Data files:**

Supplementary Data 1. Association results of all area markers across area measures in the Icelandic discovery samples.

Supplementary Data 2. All variants meeting genome-wide significance across the five DXA bone area traits

Supplementry Data 3. Association results for all area markers across area measures in the replication samples split by European and East Asian descent.

Supplementry Data 4. Association results for all area markers across all area measures in the discovery samples and replication samples combined.

Supplementary Data 5. Association results for variants reported to associate with hip shape models in Baird et al., JBMR, 2018

Supplementary Data 6. Summary of functional annotations of DXA associated markers

Supplementary Data 7. Gene-set enrichment analysis for association loci with P-value < 1e-5; showing top 15 results for each of the five phenotypes A) Femoral neck area, B) Hip, intertrhocanteric area, C) Hip, total area, D) Hip, trochanter area; FDR < 0.05 in marked blue.

Supplementary Data 8. Tissue enrichment analysis for association loci with P-value < 1e-5; showing top 15 results for each of the five phenotypes A) Femoral neck area, B) Hip, intertrhocanteric area, C) Hip, total area, D) Hip, trochanter area; FDR < 0.05 in marked blue.

Supplementary Data 9. Association of DXA area markers with joint endophenotypes in the Rotterdam samples
